# Recent advances in the biodegradation of polyethylene terephthalate with cutinase-like enzymes

**DOI:** 10.3389/fmicb.2023.1265139

**Published:** 2023-10-02

**Authors:** Beibei Sui, Tao Wang, Jingxiang Fang, Zuoxuan Hou, Ting Shu, Zhenhua Lu, Fei Liu, Youshuang Zhu

**Affiliations:** ^1^School of Biological Science, Jining Medical University, Jining, Shandong, China; ^2^Rizhao Administration for Market Regulation, Rizhao, Shandong, China; ^3^College of Chemical and Biological Engineering, Zhejiang University, Hangzhou, Zhejiang, China

**Keywords:** biodegradation, cutinase, poly(ethylene terephthalate) (PET), PETase, protein engineering, rational design, metabolic engineering

## Abstract

Polyethylene terephthalate (PET) is a synthetic polymer in the polyester family. It is widely found in objects used daily, including packaging materials (such as bottles and containers), textiles (such as fibers), and even in the automotive and electronics industries. PET is known for its excellent mechanical properties, chemical resistance, and transparency. However, these features (e.g., high hydrophobicity and high molecular weight) also make PET highly resistant to degradation by wild-type microorganisms or physicochemical methods in nature, contributing to the accumulation of plastic waste in the environment. Therefore, accelerated PET recycling is becoming increasingly urgent to address the global environmental problem caused by plastic wastes and prevent plastic pollution. In addition to traditional physical cycling (e.g., pyrolysis, gasification) and chemical cycling (e.g., chemical depolymerization), biodegradation can be used, which involves breaking down organic materials into simpler compounds by microorganisms or PET-degrading enzymes. Lipases and cutinases are the two classes of enzymes that have been studied extensively for this purpose. Biodegradation of PET is an attractive approach for managing PET waste, as it can help reduce environmental pollution and promote a circular economy. During the past few years, great advances have been accomplished in PET biodegradation. In this review, current knowledge on cutinase-like PET hydrolases (such as TfCut2, Cut190, HiC, and LCC) was described in detail, including the structures, ligand–protein interactions, and rational protein engineering for improved PET-degrading performance. In particular, applications of the engineered catalysts were highlighted, such as improving the PET hydrolytic activity by constructing fusion proteins. The review is expected to provide novel insights for the biodegradation of complex polymers.

## 1. Introduction

Poly(ethylene terephthalate) (PET) is used extensively worldwide in plastic products, and around 350 million tons have been produced in 2017 (Geyer et al., 2017). However, the accumulation of PET waste and its resistance to degradation in the environment have posed a serious threat and become a global concern (Choi et al., 2022; Li et al., 2022). PET has different crystallinities according to its usage, which leads to PET materials with different features and chemical properties, such as the fluctuation of polymer chains, surface area, functional groups, molecular weight, hydrophobicity, and melting temperature (Kawai et al., 2019). This has created another problem for scientists attempting to develop a strategy to efficiently degrade all kinds of PET wastes (Thomsen et al., 2022a).

To date, several strategies have been proposed for recycling PET waste, including primary recycling, mechanical recycling, chemical recycling, energy recycling, and biological recycling (Cao et al., 2022). Enzyme-based hydrolysis of postconsumer plastic (biological recycling) is an emerging strategy for closed-loop recycling of PET. This is a process catalyzed by PET-degrading enzymes (or microorganisms) that breaks the PET polymer into diverse products, such as terephthalic acid (TPA), ethylene glycol (EG), bis-(hydroxyethyl) terephthalate (BHET), and (mono-(2-hydroxyehyl)terephthalic acid (MHET)). This is the interactive result of active site accessibility to the polymer surface (Zumstein et al., 2017), and especially it was found that the endo-lytic activity (ability to hydrolyse internal bonds in the polymer) was a key parameter for overall enzyme performance (Schubert et al., 2023). Therefore, a circular carbon economy for PET is theoretically attainable through rapid enzymatic depolymerization followed by directed conversion into other high value-added products (Ru et al., 2020; Ellis et al., 2021; Kakadellis and Rosetto, 2021; Simon et al., 2021).

In a broad sense, the PET-degrading enzymes can be divided into the following categories according to their different functions: surface-modifying enzymes and PET hydrolases (Kawai et al., 2019). The former is usually applied for the enzymatic surface modification of polyester fibers to improve hydrophilicity (surface hydrophilization), including lipases, carboxylesterases, and cutinases (Biundo et al., 2018). These enzymes usually act on the ends or the loop (defined as a polymer chain, both ends of which are attached to the PET surface) on their surface of polymer chains, which are then hydrolysed to carboxylic acid and hydroxyl residues, increasing surface hydrophilicity (Kawai et al., 2017; Oda et al., 2018). The latter could significantly degrade the inner block of PET, as revealed by visible changes found through SEM. In addition, PET hydrolases are capable of performing surface hydrolysis, which would lead to the hydrolysis of the surface polymer chain instead of degrading the building blocks of PET.

The first enzymatic PET degradation study was reported by Rolf Joachim et al. (2005). It was shown that the obtained hydrolase hydrolysed two kinds of PET films at 55°C. Soon afterwards, the first purified poly(butylene terephthalate-co-adipate) (BTA) hydrolase 1 was purified and characterized from the culture supernatant of *Thermobifida fusca* (*T. fusca*) (Kleeberg et al., 2005), which further revealed that the sequence of *Tfu0883* is consistent with that of the gene for BTA-1 (Lykidis et al., 2007). Since then, various effective enzymes have been observed to perform enzymatic PET hydrolysis. These enzymes with PET-degradation activity mainly belong to the subfamily of carboxylic ester hydrolases (EC 3.1.1.x), including carboxylesterase (EC 3.1.1.1) (von Haugwitz et al., 2022), lipases (Moyses et al., 2021), cutinases (EC 3.1.1.74) (Wei and Zimmermann, 2017), and arylesterase (EC 3.1.1.2) (Austin et al., 2018). In addition, several PETase-like enzymes have been identified, extending the scope of enzymatic PET recycling (Almeida et al., 2019; Karunatillaka et al., 2022; Li et al., 2022). Among them, great achievements have been accomplished in the fields of crystallographic and protein engineering of *T. fusca* cutinases with various released structures [e.g., PDB ID: 4CG1 (Roth et al., 2014), 6EQH (Austin et al., 2018), 6AID (Kitadokoro et al., 2019), 7VPB (Li et al., 2022)], rational protein engineering (Lu et al., 2022), and metabolic engineering or synthetic biology-driven studies (Brandenberg et al., 2022; Jia et al., 2022). Examples include the TfCut2 variant (Wei et al., 2016), Cut190 variant (Oda et al., 2018), HiC (Ronkvist et al., 2009), and leaf-branch compost cutinase (LCC) (Sulaiman et al., 2012; Xue et al., 2021). In particular, LCC was shown to be among the most effective enzymes and has a potential ability to recycle PET. The engineered LCC was shown to breakdown a minimum of 90% of PET into monomers over 10 h (Tournier et al., 2020). There are several excellent reviews or studies illustrating the molecular engineering, applications of cutinases and other biological strategies for PET hydrolysis (Zumstein et al., 2017; Ellis et al., 2021; Tamoor et al., 2021; Urbanek et al., 2021; Kumar et al., 2022; Brackmann et al., 2023).

In 2016, a novel bacterium, *Ideonella sakaiensis* 201-F6, was reported to proliferate on a low (1.9%)-crystallinity [The degree of PET crystallinity (X_C_) indicates the fraction of the total polymer chains being in the crystal structure state (Thomsen et al., 2022b)]. PET film at 30°C for 40 days and hydrolyse PET with catalysis by the two released enzymes [ISF6_4831 (*Is*PETase) and ISF6_0224 (*Is*MHETase)] (Yoshida et al., 2016). This led to the rapid development of *Is*PETase, an *Is*PETase-like enzyme, in the field of PET biodegradation. In addition, based on modern biotechnology, such as deep sequencing technologies, metagenomics, and AI-based bioscience, novel hydrolases with PET-degrading activity have been discovered and characterized, such as Ple629 (Meyer-Cifuentes et al., 2020), Mors1 and OaCut (Blázquez-Sánchez et al., 2022), MG8 (Eiamthong et al., 2022), RgPETase (Sagong et al., 2021) and *Bb*PETase (Sagong et al., 2022).

To date, about 89 different enzymes [PAZy database (Buchholz et al., 2022)^1^] with PET-degrading ability have been discovered and characterized (Magalhães et al., 2021), and with the rapid development of bioinformatics and machine learning, an increasing number of PET hydrolases have been sourced from natural diversity (Erickson et al., 2022). This greatly expands the number and diversity of efficient scaffolds for enzymatic PET deconstruction. All effective PET hydrolases might belong to the family of cutinases or homologous enzymes (Kawai et al., 2019). Although the application of PET hydrolase is a green and sustainable strategy for PET degradation, it has been hampered by the lack of robustness to pH and temperature ranges, slow reaction rates and inability to directly use untreated postconsumer plastics. Therefore, research to improve the stability of PET-degrading enzymes and designing robust industrial catalysts is still facing great difficulties. It is worth noting that several biotech companies have long been committed to transforming plastic waste into recycled and recyclable plastic, such as the French company Carbios^2^ and the commercial hydrolase enzymes from Novozymes [e.g., CalB (Lipozyme^®^ CALB L), RmL (Palatase^®^ 20000L), CaL (Eversa^®^ transform), TlL (Lipozyme^®^ TL), HiC (Novozym^®^ 51032)].

In this study, a brief overview of the recent advances in special cutinases and cutinase-like enzymes with PET-degrading activities is provided, including their structures, catalytic mechanisms, and applications. In particular, rational protein engineering based on macromolecule structures is highlighted. Finally, further perspectives on the industrial applications of enzyme-catalyzed PET degradation are also discussed.

## 2. Promising enzymes for PET degradation

Considering that polymer molecules tend to become flexible at temperatures above the glass transition temperature (*T*_g_), the high *T*_g_ value of PET makes the thermostability of PET-degrading enzymes crucial for the efficient depolymerization of PET (Kawai, 2021). It is believed that the optimal temperature of a desired PET hydrolase needs to be >60 or preferably >70°C. Based on this recognition, a few thermophilic enzymes, including the TfH variant (TfCut2) (Mrigwani et al., 2023), LCC variant (LCC^ICCG^) (Tournier et al., 2020), Cut190 variant (Kawai et al., 2022), HiC (Carniel et al., 2021), and BhrPETase (Xi et al., 2021), satisfy the requirements for thermostability above 60°C. In addition to thermophilic enzymes, the discovery of *Is*PETase from the mesophilic bacterium (*Ideonella sakaiensis*) caused mesophilic PET-hydrolysing enzymes to become a research hot spot. In this section, recent developments in special PET hydrolase with bright prospects are highlighted (Table 1).

**Table 1:** Promising catalysts with PET-degrading activity.

Catalysts	Enzymes	The variants	References
Cutinase from bacteria	Cut190	Cut190 ^(L136F/Q138A/S226P/R228S/D250CE296C/Q123H/N202H/K305del/L306del/N307del)^	Kawai et al. (2022)
	LCC	LCC^ICCG^ (LCC^F243I/D238C/S283C/Y127G^)	Tournier et al. (2020)
	*Tf*Cut1/*Tf*Cut2	*Tf*Cut2^G62A/F249R^, *Tf*Cut2 ^S121P/D174S/D204P^	Mrigwani et al. (2023) and Li et al. (2022)
	BhrPETase	BhrPETase^H184S/F93G/F209I/S213K^	Chen et al. (2022)
	Est119/Est1	Est119^H248S/Q131G/F248I/I252K^, Est1^A68V/T253P/M259K^	Chen et al. (2022)
Cutinase from fungi	HIC		Carniel et al. (2021)
	FsC	FsCL^182A^, SDFsC	Hellesnes et al. (2023) and Zhu and Wei (2019)
	AbC	AbC^A84F^	Hellesnes et al. (2023)
	AfC	/	Ping et al. (2017)
	AoC	AoC^L185N^	Shirke et al. (2017)
Metagenome- derived PET hydrolase	PES-H1/PES-H2	PES-H1^L92F/Q94Y^	Wolfgang et al. (2019)

(/: not given).

The best enzymatic degradation reactions involving PET are carried out at (or near) PET’s glass transition temperature (~70°C), the temperature at which chains become mobile and accessible to the active site(s) of thermophilic hydrolases (Kawai, 2020). The following enzymes are notable due to their ability to function at temperatures between 60°C and 70°C (Sonnendecker et al., 2022): (i) metagenomically derived leaf branch compost cutinase (LCC) (Zheng et al., 2022); (ii) *Humicola insolens* cutinase (HiC) (Carniel et al., 2021); (iii) *Thermobifida fusca* cutinase (TfCut2) (Mrigwani et al., 2023); (iv) PES-H1 (or polyester hydrolase 7, PHL7), a close homolog of LCC that is twice as active as LCC (Richter et al., 2023); and (v) BhrPETase (from bacterium HR29), another close homolog of LCC that shows comparable activity.

### 2.1. Catalytic mechanisms of PET hydrolase

Computational studies on the catalytic mechanism of PETase and LCC were performed using quantum mechanics/molecular mechanics (QM/MM) MD simulations with different algorithms (Boneta et al., 2021; Zheng et al., 2022). Based on the free energy surfaces, the established four-elementary-step mechanism was further confirmed, including mainly (1) the acylation of the serine of the catalytic triad, (2) breakage of the C-O bond, (3) nucleophilic attack by water molecules, and (4) deacylation. It was also revealed that the similar catalytic behavior of the active site might lead to a similar catalytic mechanism, and the better performance of LCC^ICCG^ than PETase might result from the higher optimal working temperature and the special structure–activity relationships with the PET polymer (Boneta et al., 2021).

### 2.2. Cutinase

Cutinases can usually hydrolyse both ester bonds found in aliphatic and aromatic polyesters, which display very large potential for biocatalytic plastic recycling (Carr et al., 2020; Ribitsch and Guebitz, 2021). In the hydrolysing process catalyzed by cutinases, the active-site serine residue is acylated by the substrate. Then, the substrates are transformed to carboxylic acids and alcohols through the formation of an acyl enzyme intermediate. This serine residue is located within a highly conserved GXSXG sequence motif and forms a catalytic triad with His and Asp.

#### 2.2.1. Cut190 and the related variants

**Figure 1:**
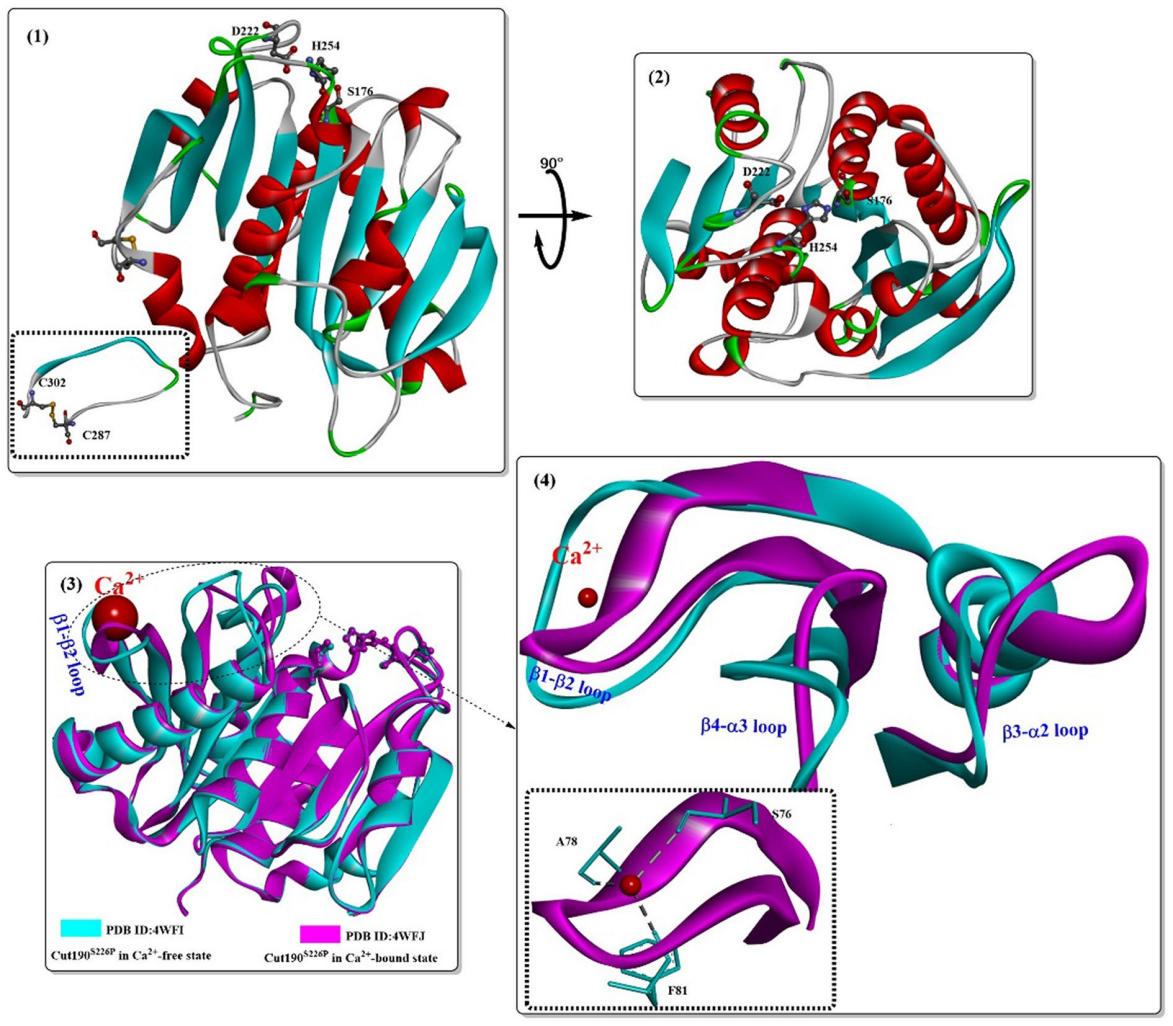
Structures of the Cut190 mutant (1,2; PDB ID: 4WFI) and the structure alignments of the Ca^2+^-free state and the Ca^2+^-bound state (3,4; PDB ID: 4WFJ). [The figures in this manuscript were generated using the program Discovery Studio 3.0; the special structural elements are shown in ball-and-stick model; and the dotted green (or light green) lines indicate the formed H-bonds].

Oda (2021) provided a great contribution to the development of Cut190. In 2014, a putative cutinase gene (*cut190*, from the thermophile *Saccharomonospora viridis* AHK190) was cloned and successfully expressed (Kawai et al., 2014). The mutant Cut190 (S226P/R228S) was shown to display optimal activity at 65–75°C, pH 6.5–8.0, and could remain stable at temperatures up to 65°C for 24 h; however, 60% of activity was lost after incubation for 1 h at 70°C. In particular, Ca^2+^ could clearly change the tertiary structure of Cut190, and the thermostability and activity could be greatly enhanced by high concentrations of calcium ions. To illustrate the structural basis for the Ca^2+^-enhanced thermostability, Takuya et al. reported the crystal structures of Cut190^S226P^ in the Ca^2+^-bound and free states (Figure 1(1,2)) (Miyakawa et al., 2015). It mainly adopts a classical α/β hydrolase fold and contains 9 β-strands, 6 α-helices and a disulfide bond linking C287 and C302. The active-site Ser-His-Asp catalytic triad (S176, D222, and H254) was located on the surface without the lid structure. Ca^2+^ was located at the β1-β2 loop (Figure 1(3,4)) formed by residues from S76 to F81 (Figure 1(4)). Furthermore, the bound Ca^2+^ was coordinated by S76, A78, and F81 via their main-chain carbonyl groups. Once Ca^2+^ bound to Cut190^S226P^, it induced significant conformational changes, which mainly occurred in the following loops: the β1-β2 loop, the β3-α2 loop (F97-A121), and the β4-α3 loop (I126-E155). Upon Ca^2+^ binding, it will induce N133 to move toward the Ca^2+^ binding site (the β1-β2 loop, ~11.5Å), consequently enabling the movement of Q110 toward D131 and forming an additional hydrogen bond. The β3-α2 loop harbors the active-site sealing residue F106. Although Ca^2+^ binding induced the β3-α2 loop to the flexible open state, it might be helpful for substrate binding.

**Figure 2:**
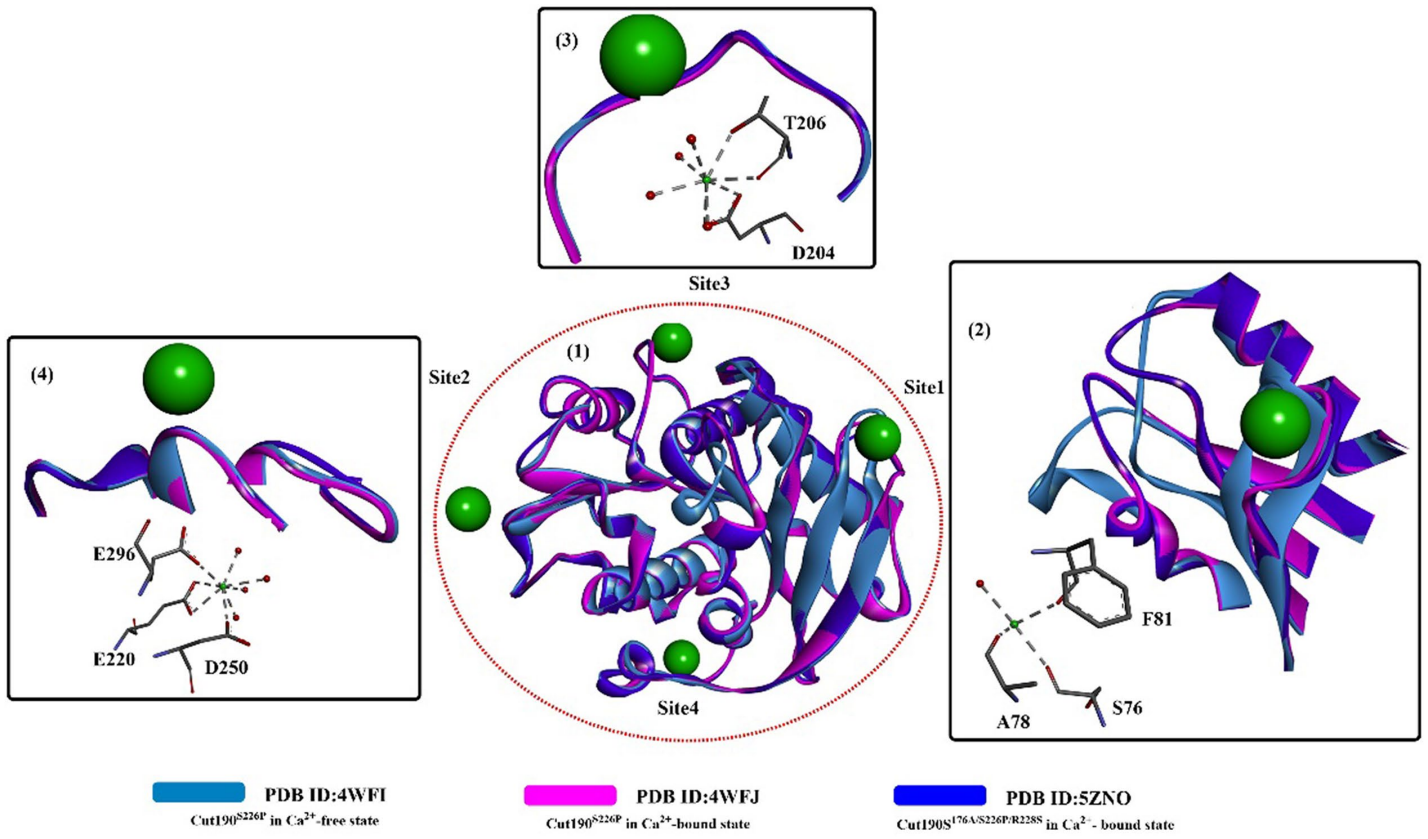
The structure alignment of the Ca^2+^-free state and the Ca^2+^-bound states (PDB ID: 4WFJ, PDB ID:5ZNO) (1) of Cut190^S176A^ and the interactions of specific Ca^2+^ with local critical residues in the different sites (2,3,4). [The ball-and-stick models below in 2,3,4 mean the interactions formed and the dotted green (or light green) lines indicate the formed H-bonds].

Based on these findings, attention was focused on insights into the structure–activity relationship of substrate-Cut190 and rational protein engineering. Molecular modeling (*ghecom*: pocket finding; *fkcombu*: docking; *the myPresto program package*: energy minimization) was applied to investigate the reaction mechanism and improve the enzyme activity by Kawabata et al. (2017). The results showed that W201, F106, H175, M177 and F255 are important for substrate binding and enzymatic activities, especially for the aromatic polyester substrate. Numoto et al. (2018) further analyzed the substrate recognition mechanism of Cut190 with the crystal structure of the inactive Cut190 mutant (Cut190^S176A^). In this study, it was found that four Ca^2+^ ions were bound to Cut190^S176A^ (Figure 2), which is different from that observed in a previous study (Miyakawa et al., 2015). From Figure 2, three of them are bound to the surface of the enzyme at site 1, site 2 and site 3. One more bound Ca^2+^ is located at the interface in site 4, which might be caused by the crystal packing that stabilizes the crystalline state of the protein. Structure alignment shows that site 1 is the same Ca^2+^ binding site, and Ca^2+^ binding displays a similar effect on the protein as that observed in the structure of Cut190^S226P^ (Miyakawa et al., 2015) (Figure 2(1,2)). At site 2, G80 moved slightly away from the Ca^2+^-binding site upon Ca^2+^ binding, and Ca^2+^-binding showed little effect on site 3 (Figure 2(3,4)). Researchers reported that functions for the different Ca^2+^ are believed to be different, in which the Ca^2+^ at sites 1 and 3 might be responsible for the regulation of enzymatic activity, and Ca^2+^ at sites 2 and 3 can accommodate thermostability. It was further verified that Mn^2+^ and Mg^2+^ probably bind to site 2 with high affinity (responsible for stability improvement) and to sites 1 and 3 with low affinity (responsible for activity adjustment) (Senga et al., 2019). These results indicate that it might be possible to regulate the catalytic activity and thermostability by rational engineering of critical residues around site 1, site 2 and site 3.

**Figure 3:**
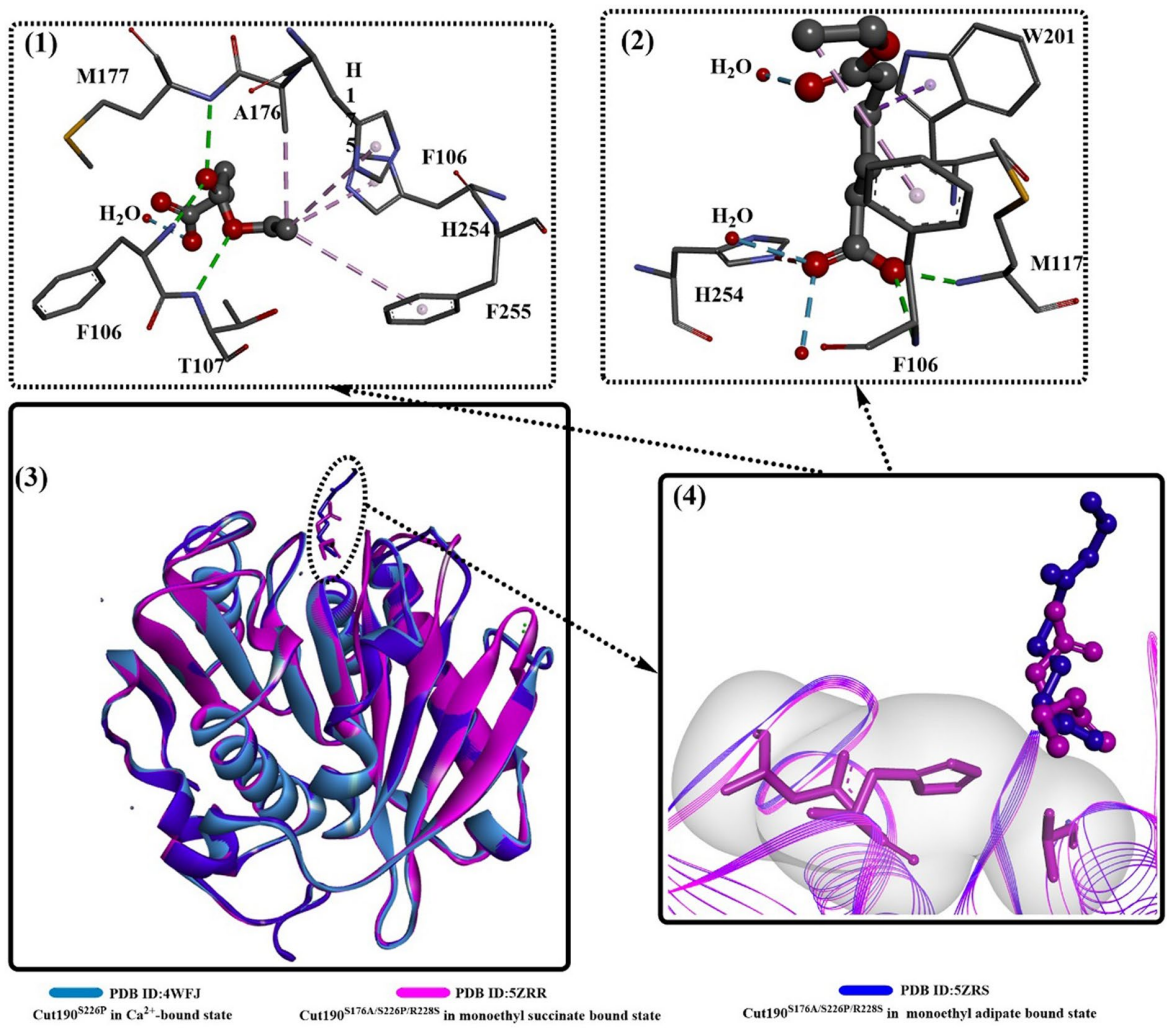
Structure alignment of different substrates (3), the local detailed structures (4) and the interaction modes of Cut190^S176A^ with monoethyl adipate (1, open forms, PDB ID: 5ZRS) and monoethyl succinate (2, engaged forms, PDB ID: 5ZRR). [The dotted pink lines mean the formed hydrophobic interactions, the dotted green (or blue) lines indicate the formed H-bonds or weak H-bonds, and the line ribbons indicate the protein model].

Structure comparison of Cut190^S176A^ bound with different substrates (Figure 3) clearly shows that two distinct states occur in the catalytic process, the prereaction state (engaged form, PDB ID: 5ZRR) and postreaction state (open form, PDB ID: 5ZRS). Interestingly, the structure of the postreaction state is almost identical to the structure bound with only one Ca^2+^. This might indicate that Ca^2+^ could greatly accelerate from the closed and the engaged form to the open form, which could also be illustrated by the weak Ca^2+^ binding interaction in site 1.

**Figure 4:**
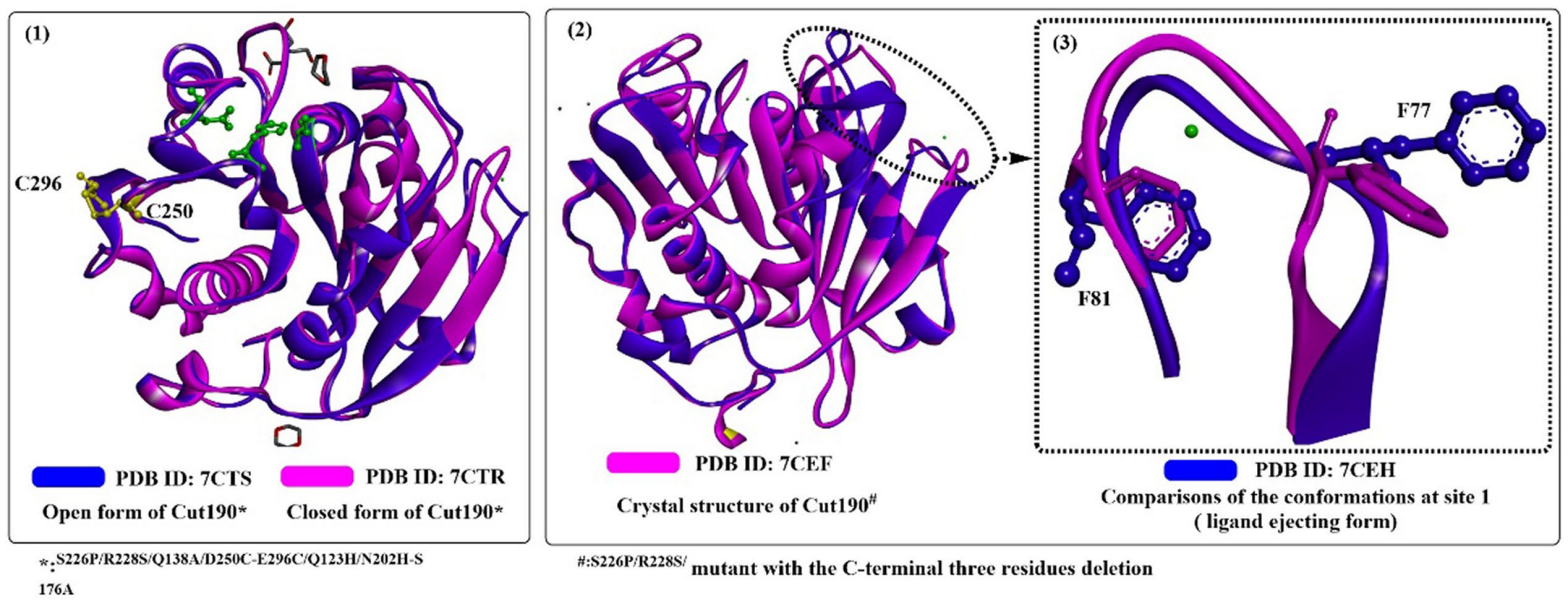
The structure alignment of Cut190*SS and Cut190*SS_S176A (1; PDB ID: 7CTR, PDB ID: 7CTS), Cut190** and Cut190**S176A (2; PDB ID: 7CEF, PDB ID: 7CEH) and the ejecting form of Cut190*(3). (The green residues mean the catalytic triads, and the yellow resides mean the introduced disulfide bond).

Recently, it was reported that mutating one Ca^2+^-binding sites of Cut190^D250C/E296C^ would increase its thermostability while causing little effect on its polyesterase activity (Emori et al., 2021). First, a disulfide bond would be introduced at the interface through the mutation of D250C/E296C (in site 2) without changing the overall structures (Figure 4(1)), which contributes greatly to the increase in thermostability. Furthermore, due to the weak binding of Ca^2+^ in site 1, the catalytic activity was unaffected when the conformational change was altered from the engaged state to the open state. The Ca^2+^ not bound to sites 2 and 3 is mainly caused by mutations in site 2 (D250C and E296C) and the residue nearby site 3 (N202H), which also reveals that Ca^2+^ binding has little effect on its overall structure (Figure 4(2)). Rapid release of the degrading products is a critical process for efficient PET-degrading, and this process has been already determined in the open form of Cut190 through molecular dynamics simulations (Numoto et al., 2018). One possibility is that the final products might be transported back to the engaged form and be tightly regulated. To deal with this issue, recently a novel state, the ejecting form at site 1 (Figure 4(3)) in the Cut190*-mediated catalytic process was determined with three C-terminal residues deleted in complex with metal ions (Senga et al., 2021). The closed-like and ligand-incompatible form displays a special conformation of F81 compared with the previous study (Numoto et al., 2018), in which either F77 and F81 all exhibit the inward conformations despite in the absence of bound Ca^2+^. This form is determined to allow the products to irreversibly dissociate, which should be properly controlled in the presence of an appropriate concentration of Ca^2+^. This phenomenon could also be illustrated by the finding that weak binding affinity of Ca^2+^ plays a critical role in the function of Cut190 (Senga et al., 2019). Once the products are irreversibly released, and the next cycle of reaction would be efficiently performed. In addition, deletion of three C-terminal residues contributes to the increased stability (*T*_m_, 85°C), which is largely the consequence of the decreased fluctuation.

Recently, Cut190 (S226P/R228S) was further engineered (Kawai et al., 2022). Using the best performing Cut190 variant (L136F/Q138A/S226P/R228S/D250CE296C/Q123H/N202H/K305del/L306del/N307del) and amorphous PET powders, more than 90 mM degradation products were obtained in 3 days and approximately 80 mM in 1 day.

#### 2.2.2. LCC and the related variants

##### 2.2.2.1. Structures

**Figure 5:**
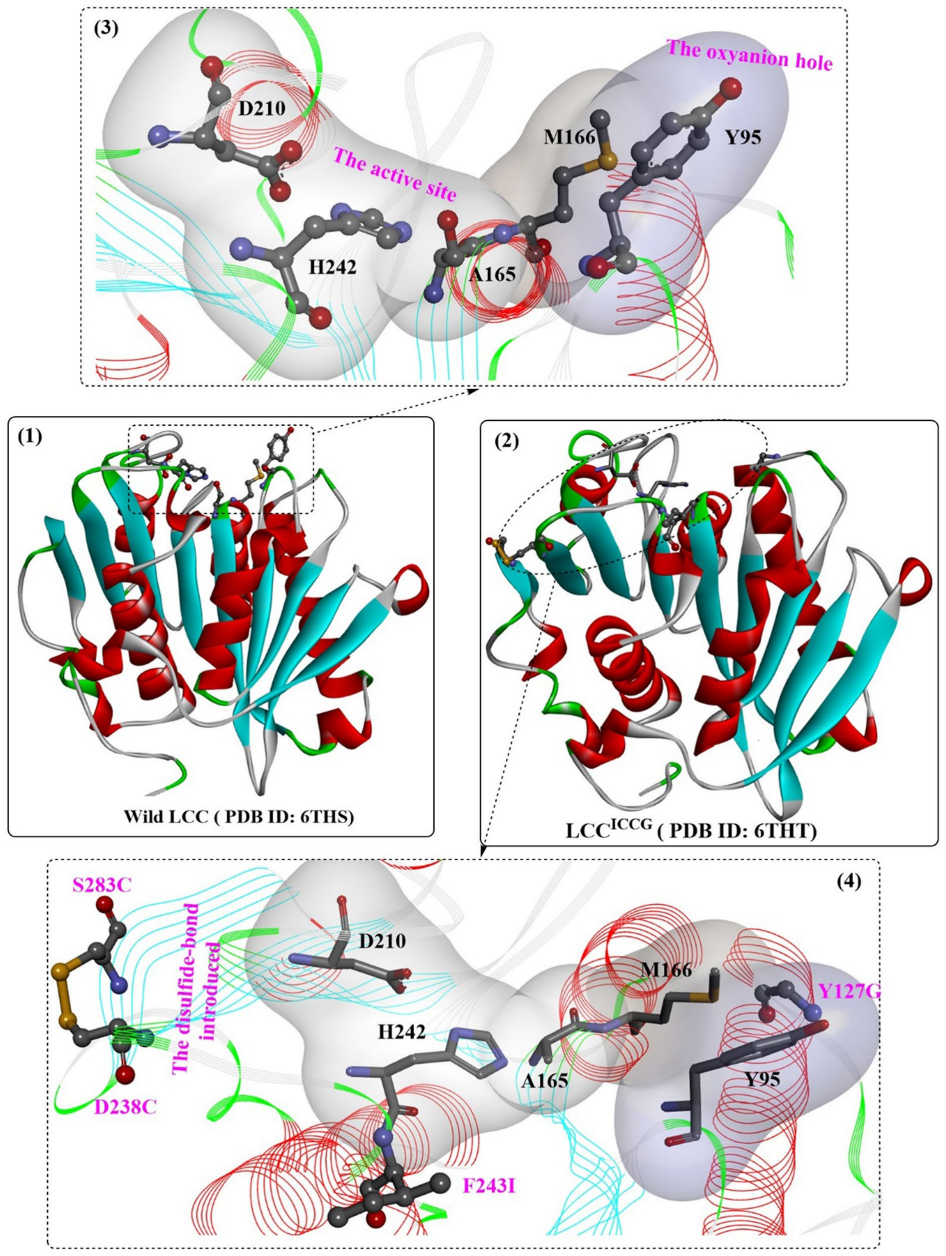
The structure of wild LCC (1), LCC ^ICCG^ (2) and the detailed structures of the substrate binding sites. (3: the local detailed structures of the catalytic triads and the formed oxyanion hole of wild LCC; 4: the detailed structures of the catalytic triads, the formed oxyanion hole and the introduced disulfide bond of wild LCC).

Sulaiman et al. (2012) reported the cloning and heterogeneous expression of a cutinase homolog, LC-cutinase, from a fosmid library of a leaf-branch compost metagenome. The enzyme shows 59.7% acid sequence identity to the *Thermomonospora curvata* (*T. curvata*) lipase and 57.4% identity to *Thermobifida fusca* (*T. fusca*) cutinase. The catalytic triad was formed by Ser165, Asp210, and His242, and an oxyanion hole (formed by Tyr95 and Met166) and a typical pentapeptide GxSxG sequence motif were also fully conserved in the LC-cutinase sequence (Figure 5(1,3)). A recent NMR study confirmed that Ca^2+^-binding site 2 (formed by D238-S283-E208, with higher affinity) and Ca^2+^-binding site 3 (formed by D193-T195, with weaker affinity) were also detected, which is similar to those of Cut190 (Charlier et al., 2022).

The enzyme could not only hydrolyse various fatty acid monoesters (with acyl chain lengths of 2–18) at pH 8.5 and 50°C but also degrade PET at pH 8.0 and 50°C with a specific degrading activity of 12 mg/h/mg of enzyme. At that time, the PET degradation rate of LC-cutinase was reported to be higher than the reported values for other cutinases by 230- to 970-fold. However, the thermostability is slightly less stable than that of *T. fusca* cutinase, with half-lives of 40 min at 70°C and 7 min at 80°C.

##### 2.2.2.2. Protein engineerings

One of the most important variants of LCC [known as ICCG-LCC, Figure 5(2,4)] was rationally designed and characterized in 2020 by Tournier et al. and contains a four-site mutant, F243I, D238C, S283C, and Y127G (Tournier et al., 2020). In this study, molecular docking and enzyme contact-surface analysis were first performed to explore the structure–function relationship between LCC and the substrate 2-hydroxyethyl-[monohydroxyethyl terephthalate)_3_(2-HE(MHET)_3_]. Around the binding site, 15 critical amino acid residues were identified, among which 11 positions were further subjected to site-specific saturation mutagenesis, resulting in 209 variants. Although most displayed impaired depolymerization activities, F243I and F243W were identified to show improved activity, and T96M, Y127G, N246D and N246M were found to exhibit an increased melting temperature. Given that the disulfide bond enhanced thermostability, the D238C/S283C variant was designed with a disulfide bond. This led to an increase in melting temperature (94.5°C) by 9.8°C compared with that of wild-type LCC, but the catalytic activity decreased by approximately 28%. Then, F243I and F243W were added into this mutant, resulting in mutants F243I/D238C/S283C (ICC) and F243W/D238C/S283C (WCC) with higher melting temperatures (6.2°C and 10.1°C increased, respectively) but no activity losses. Finally, T96M, Y127G, N246D or N246M mutations were introduced into ICC and WCC, and the results showed that mutants ICCG, ICCM, WCCG and WCCM displayed improved melting temperatures ranging from 9.3°C to 13.4°C with little effect on specific activity compared with that of wild-type LCC. In the scale-up PET recycling process, ICCG and WCCG showed the best conversion level with 82 and 85% conversion in 20 h and 15 h, respectively. Moreover, under the same conditions, 90% depolymerization could be achieved for WCCG and ICCG after 10.5 h and 9.3 h, respectively, but a higher initial rate was observed for ICCG. The obtained ICCG mutant, as one of the most promising PET hydrolases (with a mean productivity of 16.7 g·L^−1^·h^−1^ TPA), displays great potential for future PET recycling and highlights the importance of computer-aided enzyme engineering in the field of PET degradation.

**Figure 6:**
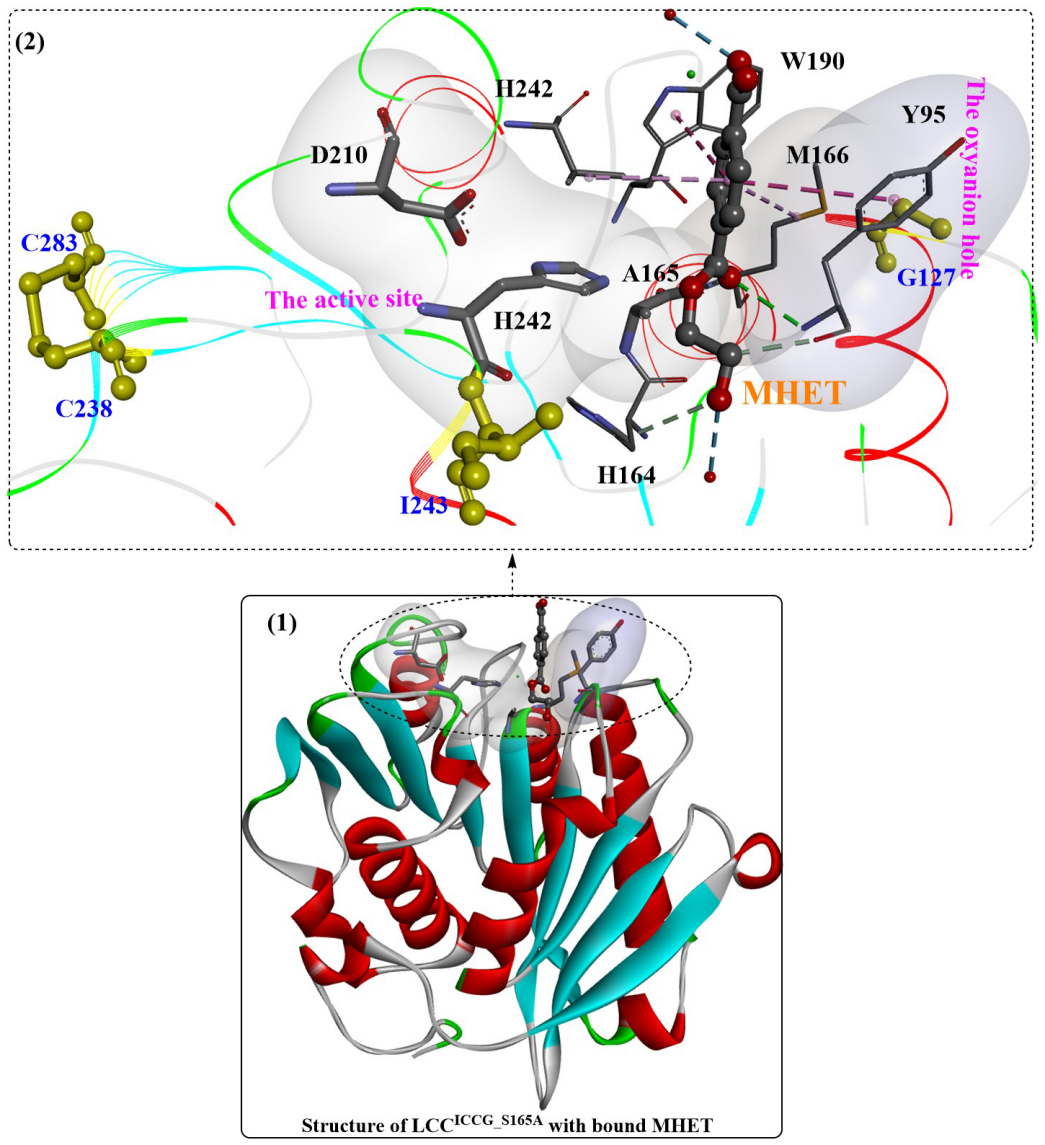
The complex structure of ICCG_S165A/MHET (1, PDB ID: 7VVE) and the interaction mode (2). [The line ribbons indicate the protein structure, the (or light) green and blue dotted lines mean the formed H-bonds, the pink dotes line mean the hydrophobic interactions and the yellow resides mean the formed disulfide bonds].

Recently, the substrate-binding mode of ICCG was investigated in detail (Zeng et al., 2022). MHET could bind to the surface cleft formed by the hydrophobic region of the active site and the oxyanion hole (Figure 6(1)). Two hydrogen bonds are formed between the carbonyl O of MHET and the main chain NH of Y95 and M166 to maintain the reaction status, and the benzene ring is further stabilized by the aromatic pi-pi interactions resulting from Y95, V212, M166 and W190 (Figure 6(2)). In addition, two water molecules are found to be associated with the formation of H-bonds with the hydroxyl at both ends of MHET. G127 and I243 (two critical mutations in ICCG) are located near the MHET-binding cleft (Figure 6(2)). G127 is located on the protein surface around the oxyanion hole region, and I243 is closer to the active site. The F243I substitution could further expand the substrate-binding site capable of increasing the PET-binding capacity, as mentioned by Tournier et al. (2020). Intriguingly, the substrate binding modes of ICCG are quite similar to those of *Is*PETase, with the different aromatic moiety conformations of the two bound ligands deviating by ∼30°.

Based on these findings, structure-based rational design was conducted to improve the thermostability of ICCG by introducing proline or hydrophilic interactions on a protein surface or increasing internal hydrophobic interactions. Among the 27 obtained variants, mutants A59H, A59R, A59K, V63I, V75R, and N248P displayed improved activity at 90°C. Considering the functional roles of N248P, N248P combined with mutants A59R, A59K, V63I, and V75R was designed to generate seven triple mutants. The results showed that all obtained triple mutants displayed higher activity than that of ICCG at 90°C. When using reinforced PET as the substrate, mutants RIP (A59R/V63I/N248P), KIP (A59K/V63I/N248P) and KRP (A59K/V75R/N248P) exhibit more effective PET hydrolytic activity at optimal operating temperatures (80–85°C). In particular, mutant RIP showed the highest efficacy at 85°C.

**Figure 7:**
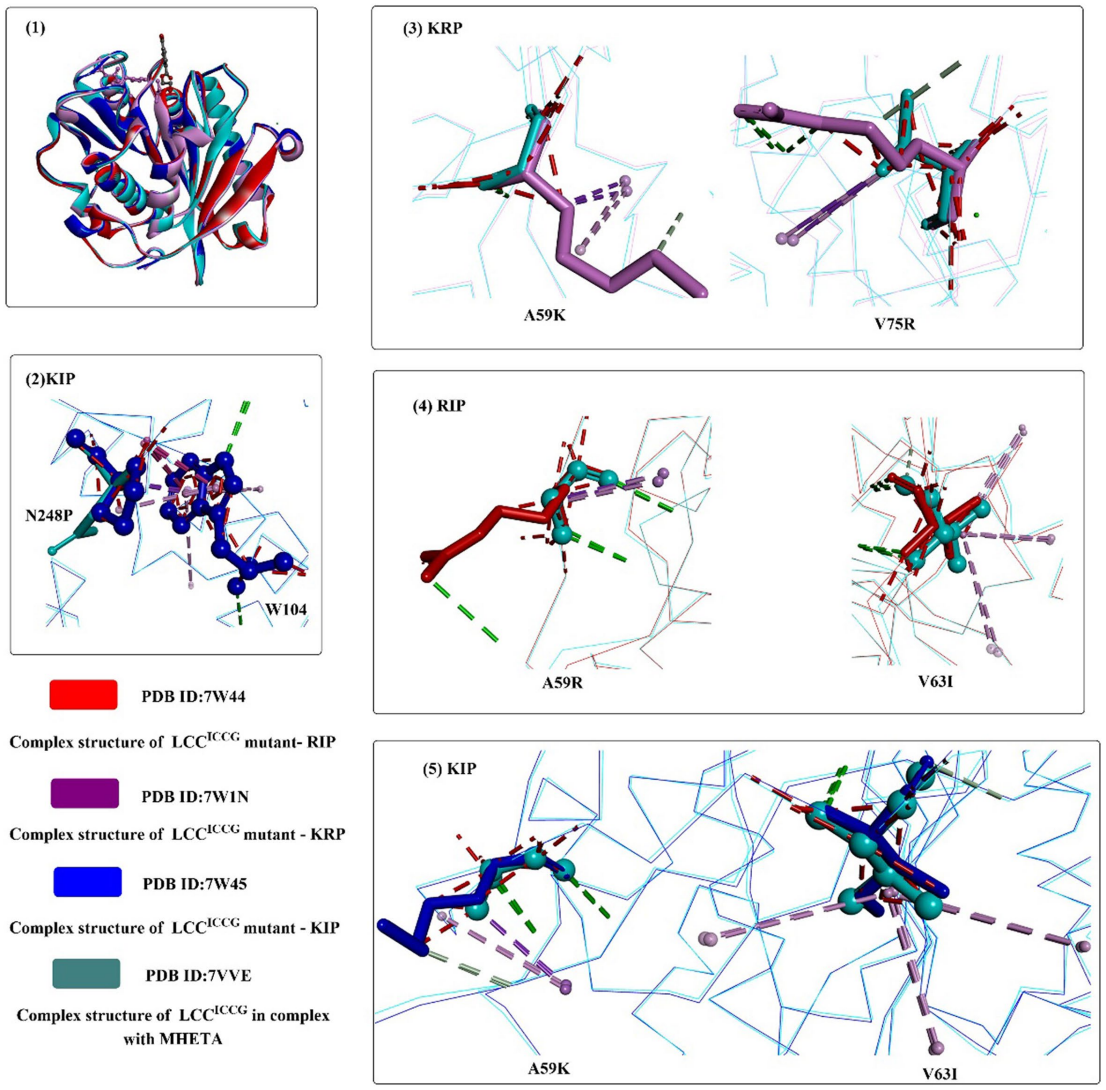
The structure alignment of LCC^ICCG-S165A^ with mutants RIP, KIP and KRP (1), and interaction modes of mutant amino acids with the surrounding residues of RIP, KIP and KRP compared with those of LCC^ICCG^ (2–5). (The mutant residues are shown in ball-and-stick models in different colors, the red dotted lines mean the steric bumps, and other dotted lines indicate the same as mentioned above).

Structure analysis showed that the crystal structures of KIP, RIP, and KRP are almost identical to the ICCG structure (Figure 7(1)), and the improved activities may be caused by the additional intramolecular interactions. N248P (located in the β8-α6 loop) could pack against the adjacent W104 to stabilize the β8-α6 loop and provide additional interactions between the loop and helix α1 (Figure 7(2)). For KRP, V75R forms two additional hydrogen bonds (shown in green line), and A59K could help form an additional hydrogen bond. Similarly, for RIP, A59R can pack against Y78 and form a hydrogen bond. The methyl group of the V63I mutant in RIP and KIP could stretch into a hydrophobic cluster sandwiched between the central β-sheet and helix α2 capable of strengthening the local hydrophobic interactions (Figure 7(4,5)). In summary, the hydrophilic interactions on the protein surface (N248P) and the intermolecular stabilization result from these mutations.

In a recent study, a semirational protein engineering approach was used to improve the catalytic activities of LCC (Pirillo et al., 2023). LCC without the secretion signal (ΔLCC) was selected as the starting target. Molecular docking and molecular simulation were used to investigate the interactions between the substrate (3PET) and ΔLCC. Based on the individual Δ*G*_bind_ values (≤ −1 kcal·mol^−1^) and the evolvability of each ΔLCC residue predicted, critical residues Y95, T96, Y127, M166, and V212 were selected as the binding hot spots capable of regulating the overall complex stability. The results obtained from site saturation mutagenesis (SSM) revealed that mutations at positions 212 and 243 lead to a significant fraction of improved variants, and F243T, F243I, V212T, V212M and T96H variants especially showed a significant increase in activity on PET nanoparticles at 50°C compared with ΔLCC (increased by ~1.75-fold, ~1.51-fold, ~1.34-fold, ~1.33-fold, and ~ 1.25-fold, respectively). For the biodegradation of PET film at 72°C, only the F243T variant showed an increased activity with a production of 21.9 mM of products with a reaction rate of 0.48 mmol_products_·mg_enzyme_^−1^·day^−1^, which is ~1.2-fold higher than that with wild-type ΔLCC. Then, mutations at A97 and S101 were introduced into ΔLCC_ F243T, which may play a role in the overall binding energy of the enzyme-PET complex. Four double variants (A97G/F243T, A97T/F243T, S101N/F243T and S101V/F243T) displaying significantly increased activity for PET degradation were obtained; furthermore, the S101N/F243T and A97T/F243T mutants showed the largest increase in activity for the degradation of PET nanoparticles (compared with ΔLCC_F243T and ΔLCC). S101N/F243T was more efficient than that of A97T/F243T mutants for the degradation of amorphous PET film at the tested temperatures and 2.0- and 3.5-fold faster than that of the wild ΔLCC at 55°C and 60°C, respectively. This study provides insights into the catalytic mechanism of LCC, especially the functional role played by F243, which should be further confirmed experimentally by crystal structures of the S101N/F243T complex.

In a very recent study (Fang et al., 2023), whole-cell screening assays combined with rational design and combinatorial mutagenesis were applied for the engineering of LCC^ICCG^. It was revealed that the mutant RITK (LCC^ICCG^-D53R/R143I/D193T/E208K) displayed better whole-cell biocatalytic performance with an 8.33-fold increase in biocatalytic activity compared to those expressing ICCG. Moreover, the thermostability was also enhanced with a 12.75-fold increase in depolymerization compared to ICCG at 85°C for 3 h. However, I believe further studies on the catalytic performance of RITK against different PET substrates with different crystallinities and the expression efficiency of different protein with the same expression vector. In a similar study, machine learning combined with evolutionary analysis was used for the rational design of LCC^ICCG^ (Ding et al., 2023). Results showed that mutants (S32L, D18T, S98R, T157P, E173Q, N213P) all displayed higher degrading-activities toward PET powder than wild LCC^ICCG^ at 75°C. Based on this finding, LCCICCG_I6M (carrying mutations S32L/D18T/S98R/T157P/E173Q/N213P) was constructed and was further determined to display the highest activity for PET powder (crystallinity 39.07%) at 75–80°C, which could release 4.028 ± 0.078 mM products with a 4.63-fold at 80°C compared with LCC^ICCG^ (0.871 ± 0.050 mM). In addition, the PET degrading performance against high crystallinity commercial PET materials (water bottle) was increased by 3.64-fold with 31.91 ± 0.99 mM soluble products detected compared with LCCICCG. Moreover, the stability was also improved with the optimal temperature increased from 65°C to 80°C. Molecular simulations suggested that N213P mutations might play a role in increasing the overall structural rigidity, and the E173Q mutation might be capable of enhancing the flexibility of the substrate-binding pocket of LCCICCG_I6M.

#### 2.2.3. *Thermobifida fusca* cutinases (TfCut)

Two open reading frames encoding cutinases named Tfu_0882 (TfCut1) and Tfu_0883 (TfCut2) were identified in the thermophilic bacterium *Thermobifida fusca* (Chen et al., 2008). Although they share a high sequence identity of 93% in the amino acid sequence (261 aa), TfCut2 displayed much higher (~ two times higher) degradation activity than that of TfCut1 (Chen et al., 2010). TfCut2 displays 57.4% similarity in amino acid sequence with LCC. The homology model of Tfu_0883 displays a typical α/β-hydrolase fold with a canonical triad S170-H248-D216, a preformed oxyanion hole and a different nucleophilic serine exposed to the solvent. Although they show similar catalytic properties to fungal cutinases, TfCut1 and TfCut2 are a different subfamily of cutinases due to their sequence and structural differences. For example, they can hydrolyse insoluble triglycerides and soluble esters (e.g., *p*NPB) in addition to cutin. They also display superior thermostability compared with that of the *F. solani pisi* cutinase. A study showed that the higher linear degradation rates observed in the first 72 h of incubation are due to the high hydrolysis susceptibility of the mobile amorphous fraction (MAF, with a chain mobility similar to the purely amorphous polymer) of PET; however, a transition of MAF to the rigid amorphous fraction (RAF, with a lower chain mobility probably caused by its presence within the intraspherulitic or interlamellar regions in close vicinity to the crystalline domains) during further incubation resulted in drastically decreased degradation rates (Wei et al., 2019). The RAF could be cleaved only in an endo-type manner at a much lower rate.

**Figure 8:**
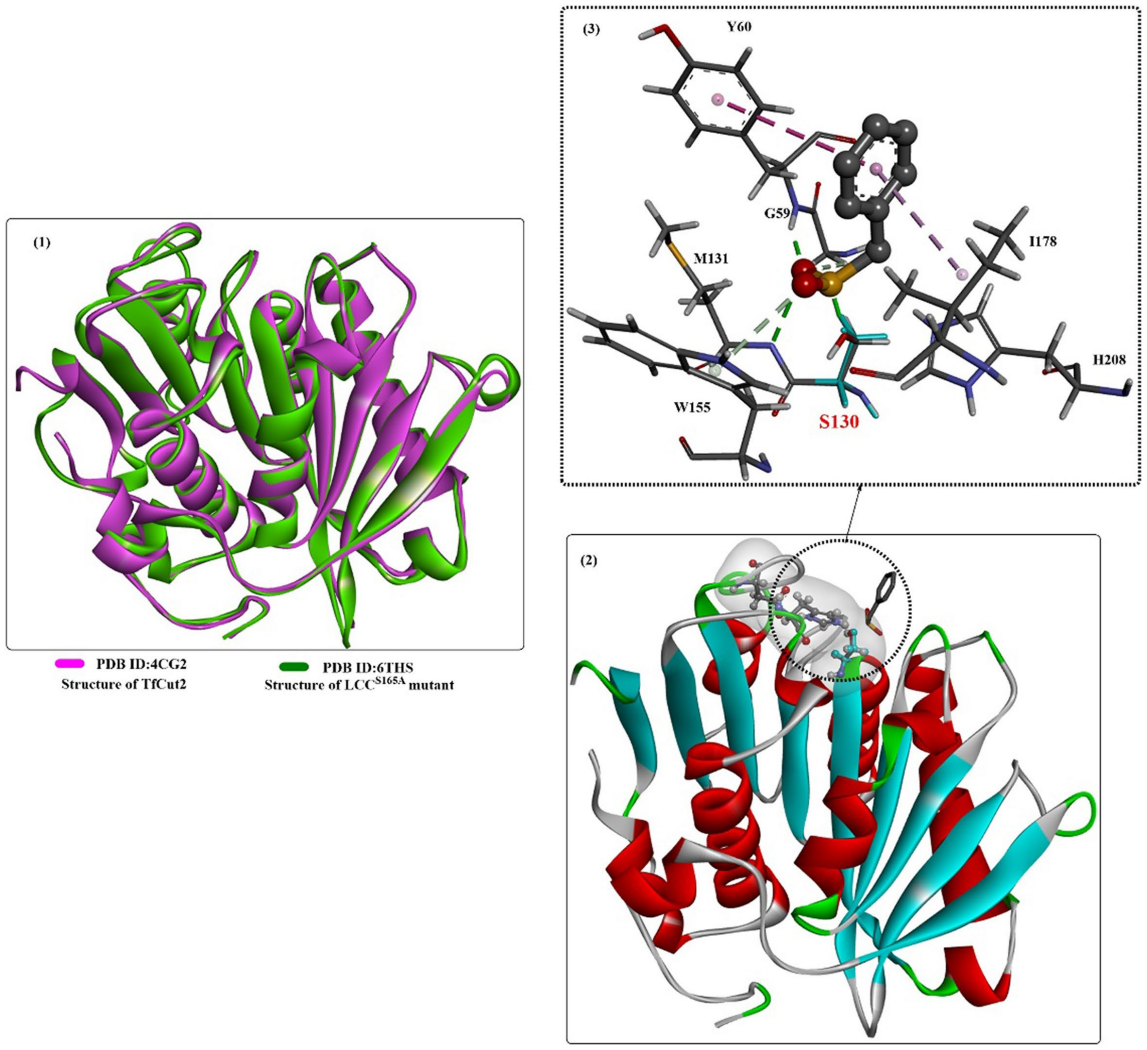
The structure alignment of TfCut2 with LCC^S165A^ (1), the crystal structure of TfCut2 with the inhibitor-phenylmethanesulfonic acid and the hydrophobic surface around the active site (2), and the local detailed interaction mode of the complex.

To investigate the catalytic mechanisms of the bacteria-derived cutinases, several crystal structures of TfCut2 have been reported [e.g., PDB ID: 4CG2 (Roth et al., 2014), Figure 8, 5ZOA, 7QJR, 7XTT]. The structure of TfCut2 forms a classical α/β-hydrolase fold, contains conserved GXSXG motif, and contains a conserved catalytic triad of S-H-D residues is in a crevice on the surface. In the enzyme-inhibitor complex structure (Figure 8(2)), it was found that covalent modification of S130 would lead to the tighter interactions formed with the main chain of M131 and Y60 mainly through H-bonds interactions (Figure 8(3)). The phenyl ring is further stabilized mainly by hydrophobic interactions with I178 and Y60 (Figure 8(2)).

Efforts have been made to further improve the catalytic performance of TfCut2 through directed evolution and rational mutagenesis. The critical residues associated with substrate recognition were engineered (Wei et al., 2016). First, critical residues around the active site close to (without direct interactions with) the docked 2PET were identified and genetically engineered by performing structure alignment with LCC and TfCut2. Four residues (G62, T63, I178, and I213) were selected for further protein engineering. Among the single mutants, G62A was determined to show markedly increased hydrolytic activity. The improved catalytic performance of G62A likely resulted from a reduction in product inhibition due to MHET rather than the altered surface hydrophobicity or the enlarged substrate binding pocket. Although the double variant G62A/I178V and the triple variant G62A/I178V/I213S showed much higher hydrolytic activity than the wild-type TfCut2, they all displayed slightly lower degrading activity than the single mutant G62A. The single mutant I213S showed the highest thermostability, followed by G62A and G62A/I213S. After 24 h at 65°C, G62A and G62A/I213S caused a 20.5% weight loss of PET films.

In a similar study, the functions of critical residues around the substrate binding site were investigated, which were thought to facilitate the accessibility of the enzyme’s active site (Dong et al., 2020). Eleven bulky residues were selected to increase the substrate binding efficiency by site-directed mutation to Ala, which are located around the catalytic triad, the oxygen hole and the substrate binding region. The results showed that Y60A, T207A, F209A, L90A and I213A exhibited increased pNPB hydrolysing activity, especially the cutin hydrolysing activities of L90A, I213A, and L90A/I213A, which were increased by 5-, 2.4-, and 3.2-fold, respectively. MD simulations revealed that the higher flexibility of the substrate binding site of mutants L90A and I213A might lead to increased hydrolysing activities toward cutin. However, a subsequent study showed that the L90A mutant showed ~11.2-fold reduced degradation activity on commercially sourced PET film, but the L90F mutation displayed considerably increased degradation (~1.5-fold increased) activity (Mrigwani et al., 2023). This might be caused by the more efficient binding of PET resulting from the replacement of the hydrophobic phenylalanine residue.

Based on the abovementioned findings, molecular rational engineering of TfCut2 was performed by docking and MD simulations to modify the space at the active site and streamline the hydrophobicity at the active site or other interacting regions (Mrigwani et al., 2023). The results showed that the selected residues H129W, W155F, H184A, and H184S for engineering the PET-binding cleft exhibited little positive effect on the catalytic activities. Residues (A173C) that were identified for the insertion of thiols/disulfides (A173C/A210C, and A173C/ A206C) might distort the PET-binding site, causing a complete loss of activity against solid PET. Interestingly, residues (L90) distal to the active site could adjust its catalytic activity, especially the L90F mutation, which could considerably increase TfCut2’s PET-degrading activity (~1.5-fold). F209I, F249A and F249R were constructed to modify the surface hydrophobicity proximal/distal to the active site. The results showed that the single mutant (F209I) caused an ~4.4-fold decrease in activity; however, F249R led to an ~1.27-fold increase in activity against solid PET. The double mutants displayed a remarkable increase in degradation activity (G62A/F209I with an ~6.4-fold improvement and G62A/F249R with an ~7.15-fold increase); however, their thermostability was reduced. These results also revealed that TfCut2 possesses high thermal stability, high kinetic-thermal stability, and high thermodynamic stability.

**Figure 9:**
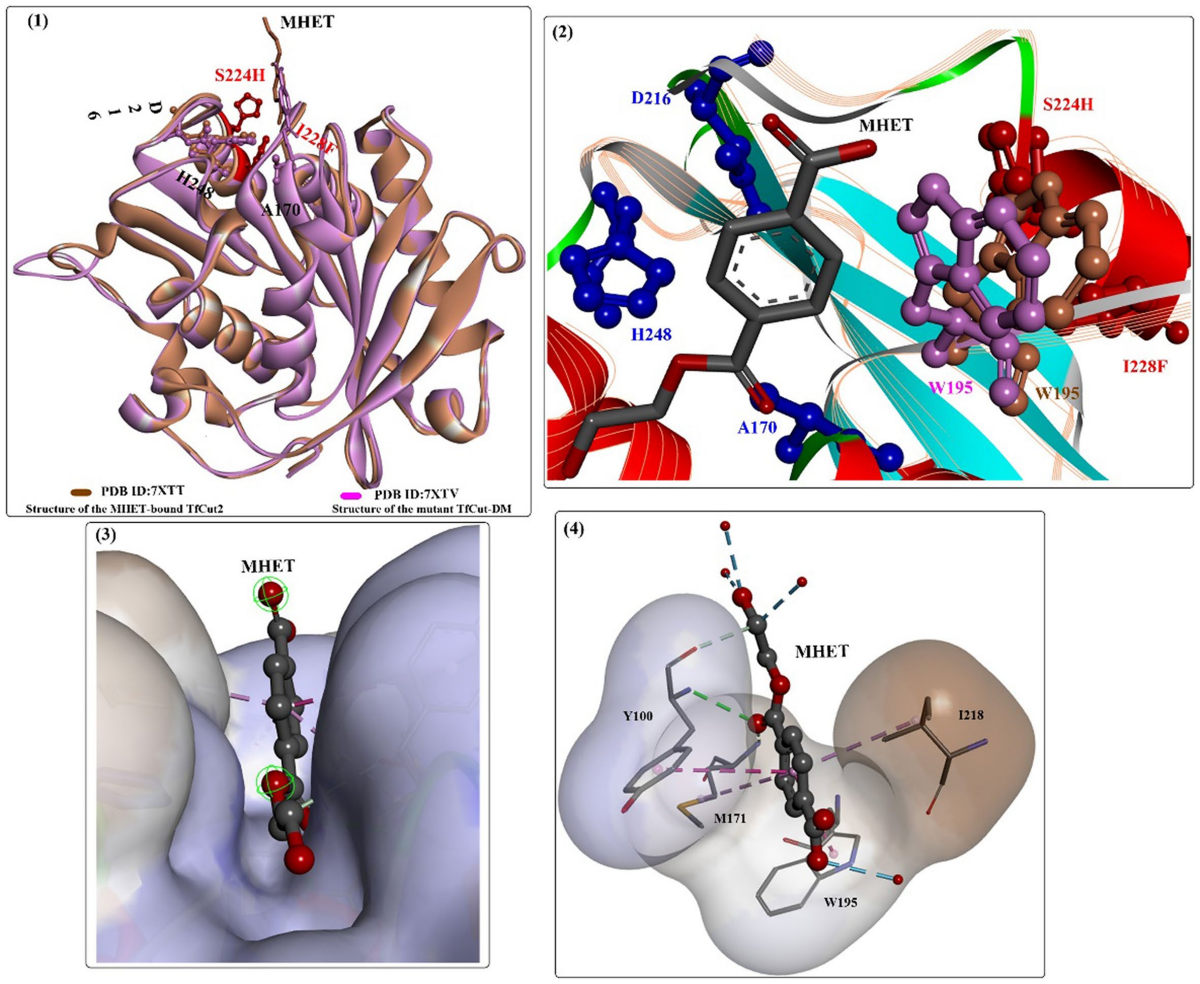
The structure alignment of MHET-bound TfCut (PDB ID: 7XTT) and TfCut-DM (PDB ID: 7XTV) (1), the different conformations of W195 associated with MHET binding (2, shown in purple and brown respectively), the binding mode of MHET in the binding cleft (3) and the detailed interaction modes (4).

Based on the findings of the double mutation (DM) strategy (Chen et al., 2021), engineered *Tf*Cut was investigated for efficient biodegradation of poly(butylene adipate-co-terephthalate) (PBAT) (Yang et al., 2023). The results showed that the mutant (*Tf*Cut-DM) is a highly potent catalyst that can completely decompose PBAT films in 36 h and release 3-fold more TPA (the major product) than TfCut within 12 h. This strategy was also determined to be appropriate for *Bur*PL and *Tc*Cut. The overall complex structure of the MHET-bound *Tf*Cut complex structure is almost identical to that of *Tf*Cut-DM (Figure 9(1)), and the DM residues (S224 and I228) are located below the catalytic triad. The ethylene glycol motif of MHET bound points outside the enzyme, and the aliphatic part stretches to fill a cleft across the protein surface (Figure 9(3)), which is lined by several critical residues, such as M171, W195, Y100, I218, and T223. Several water molecules are also shown to be associated with the binding of MHET through H-bond interactions. Interestingly, the side chain of W195 in *Tf*Cut-DM exhibits a B-type conformation [Figure 9(2) in purple]; however, wild-type *Tf*Cut harbors a TPA-binding Trp in the C-type conformation [Figure 9(2) in brown]. The DM mutation and the B-type W195 lead to a more open and flatter TPA-binding pocket (increased flexibility), which would probably cause less hindrance for the entrance of bulkier substrates. This may be the dominating factor behind the improved hydrolytic activity toward PBAT and PET.

Then et al. (2016) directly introduced a disulfide bridge (D204C-E253C) by substituting the calcium binding site of *Tf*Cut2 to eliminate dependence on calcium. The results showed that the thermal stability of the mutant was further improved, the melting point was increased to 94.7°C (*Tf*Cut2: 69.8°C), and the half-inactivation temperature was increased to 84.6°C (TfCut2: 67.3°C). Most curiously, the hydrolytic activity of the calcium-independent thermostable and efficient hydrolase was not affected when the calcium binding site was replaced. A similar study was carried out with the anchor peptide-cutinase mutant *Tf*uc2^D204C/E253C^ (Liu et al., 2022). The PET degradation rate of the obtained fused protein was determined to be up to 57.9% at 70°C for 96 h, and the catalytic performance was improved by 22.7-fold compared with that of *Tf*uc2. Based on machine learning, Li et al. successfully designed a highly thermostable *Tf*uc2 mutant, S121P/D174S/D204P, by mining molecular dynamics simulation trajectories and rational introduction of hydrogen and ionic bonds (Li et al., 2022). The results showed that the PET degradation ratio was further increased by 46.42-fold at 70°C compared to that of wild-type *Tf*Cut2.

To further increase the thermostability of the polyester hydrolases from the thermophilic actinomycete *Thermobifida fusca*, rational engineering of the binding sites of specific metal ions (e.g., Mg^2+^ and Ca^2+^) was also investigated (Then et al., 2015). Based on the MD simulations, residues Asp174, Glu253, and Asp204 of TfCut2 might play roles in facilitating the binding site of Ca^2+^, which were then mutated to screen for stability-enhanced mutants. The results showed that mutants D204R and E253R increased by more than 14°C compared to that of wild-type TfCut2; however, the PET-degrading activities were determined to be slightly reduced. In addition, it was found that the addition of a cationic surfactant (C_12_-N(CH_3_)_3_^+^) greatly improved the degradation activity of TfCut2, which might be caused by the aggregation of enzymes near the PET film surface via electrostatic interactions (Arnling Bååth et al., 2022). The results showed that in the presence of cationic surfactant (30 ppm C_12_-N(CH_3_)_3_^+^), the activity of the TfCut2^G62A/F209A^ mutant increased to 31 ± 0.1 nmol min^−1^ cm^−2^, which is approximately 12.7 times higher than that of wild-type TfCut2 in the absence of surfactant.

#### 2.2.4. BhrPETase

From the bacterium HR29, a close homolog of the LCC with 94% sequence identity was identified (BhrPETase, NCBI accession No. GBD22443) (Kato et al., 2018). Recently, high-level secretory expression of BhrPETase in *Bacillus subtilis* was successfully achieved with an expression titer of 0.66 g/L (Xi et al., 2021). More strikingly, a strong preference of BhrPETase for high temperature was determined for BhrPETase when the reaction temperature was increased from 30 to 90°C. Even at 90°C, the catalytic performance remained superior to that of wild LCC, which rapidly dropped to 55% of the maximum activity. The T_m_ value of BhrPETase was determined to be 101°C without the addition of Ca^2+^ ions, ~11°C higher than that of LCC. Against a crystallinity of 11.2% PET powder for 20 h at 70°C, approximately 6.3 mM degradation products were detected. Molecular modeling suggested that the increased thermostability might be caused by Ser175, Gln202, Pro199 and Pro248 in BhrPETase, which could either result in additional formed hydrogen bonds and/or increase the rigidity of the flexible loop.

In a recent study, molecular engineering of the hydrolase from the bacterium HR29 was reported by means of a protein language model and force-field-based algorithms (under review^3^). The obtained TurboPETase (BhrPETase^H218S/F222I/A209R/D238K/A251C/A281C/W104L/F243T^, *T*_m_ of 84°C) exhibited a 4.4-fold increase in the degrading activities toward GF-PET films compared to that of wild-type BhrPETase. Moreover, the depolymerization rate of TurboPETase (21%) outperformed all the tested PET hydrolases, which was 4-, 4-, 8- and 33-folds higher than those of wild BhrPETase, LCC^ICCG^, HotPETae and FastPETase at 65°C within 3 h, respectively. At 65°C after 24, approximately 100% depolymerization could be obtained. Especially, the full degradation of pretreated PET (up to 300 g L^−1^) could be accomplished within 10 h, with a maximum production rate of 77.3 gTPAeq L^−1^ h^−1^. It shows beyond doubt that this strategy and the obtained TurboPETase might serve as a promising option for the engineering of interfacial enzymes and efficient PET cycling.

#### 2.2.5. Est119

**Figure 10:**
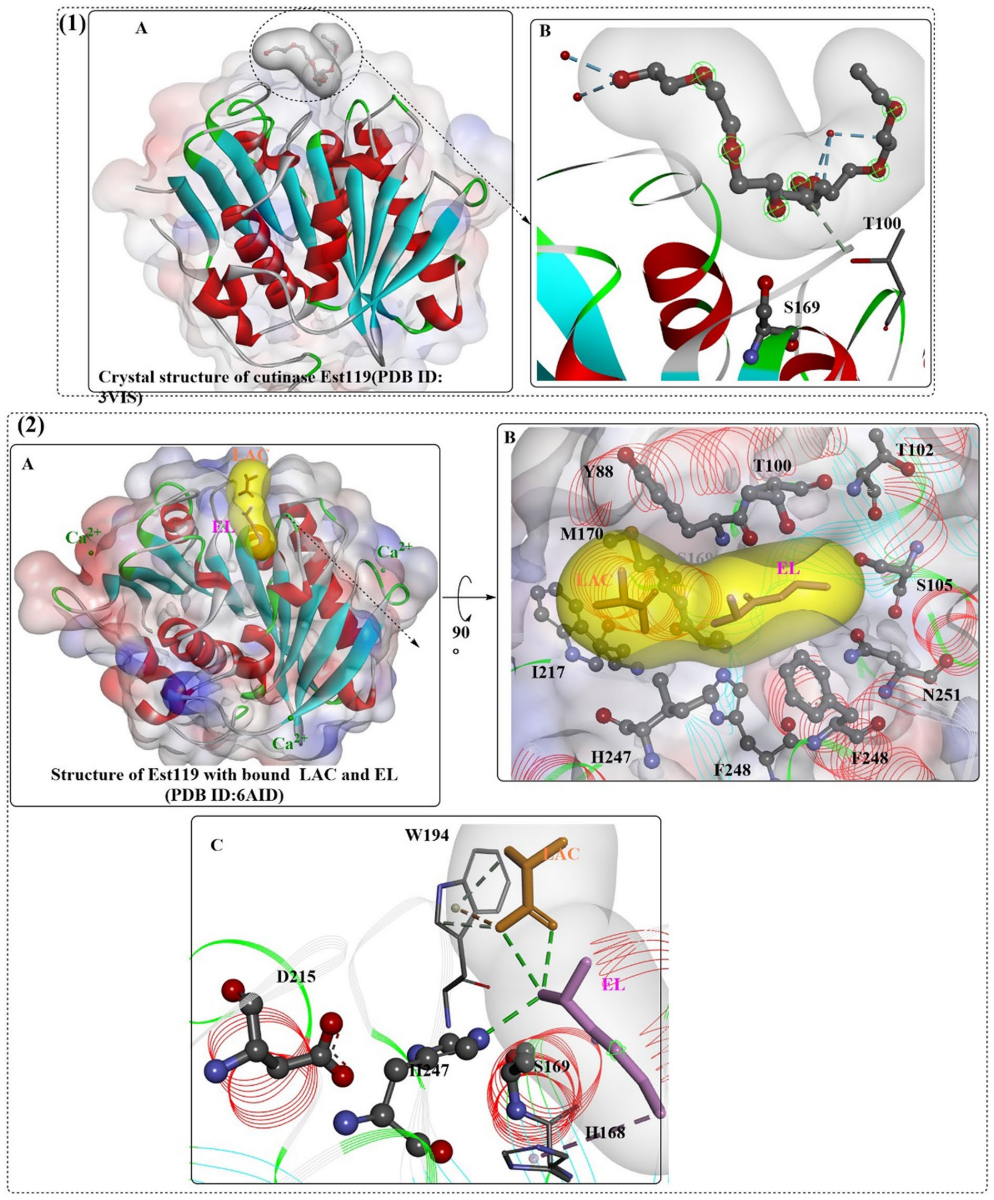
The crystal structures of Est119 with bound substrate PE4 (1A) and the local detailed interactions of the Est119- PE4 complex (1B); the crystal structures of Est119 with bound substrate LAC and EL (2A) and the local detailed interactions of the Est119- PE4 complex (2B,C). (The white and yellow surface mean the hydrophobic surfaces of the substrates in the active sites, and the colorful ones mean the hydrophobic surface of the whole protein).

*Thermobifida alba* strain AHK119 (AB298783) was shown to be capable of degrading aliphatic-aromatic copolyester film and polymer particles and releasing TPA (Hu et al., 2010). From AHK119, an esterase gene (*est119*) was cloned and identified, and the Est119 protein contains the highly conserved motif (G-X-S-X-G) of the serine hydrolase family, a catalytic triad (Ser129, His207, and Asp175) and the oxyanion hole formed by Tyr59 and Met130. The optimal reaction temperature and pH were found to be ~50°C and 6.0, respectively. Kitadokoro et al. determined the crystal structure of Est119 (Figure 10(1)), which shows a typical α/β-hydrolase fold that also consists of a central twisted β-sheet of 9 β-strands flanked by 9 α-helices on both sides (Kitadokoro et al., 2012).

In another study (Thumarat et al., 2012), it was further confirmed that Est119 is a typical cutinase-like enzyme, and Ca^2+^ was necessary to display full catalytic activity (300 mM) and maintain thermostability (200 mM, stable at 50°C for 16 h). Random mutagenesis analysis indicated that mutations with more hydrophobicity at the N-terminus would more likely result in enhanced activity, which led to mutants A68V and A68V/S219P showing the highest activity against *p*NPB. In particular, the degradation activity of A68V/S219P was further improved by ~50-fold and 1.7-fold compared with that of WT and A68V, respectively. In addition to *est119*, its homolog *est1* was located upstream of est119 (Thumarat et al., 2015). Although showing 95% sequence identity and 98% sequence similarity with Est119, Est1 displays a higher (~2-fold higher) activity toward *p*-nitrophenyl butyrate. Moreover, Est1 displayed a much higher thermostability, with 300 mM Ca^2+^, and it could maintain nearly 100% of the residual activity at 50°C for 20 h. Est1 and Est119 could also recognize the aromatic polyester and produce EG and TPA. As mentioned above, Est1^A68V/T253P^ was constructed, and the results showed that the T_m_ value was increased to 79°C and the specific activity (833 ± 22.5 U/mg) was also improved by ~57.4-fold against *p*NPB. The increased thermostability was estimated to be caused by the introduction of proline, which could decrease the flexibility and increase the structural rigidity, especially at sites 219 and 253. However, Est1^A68V/T253P/M259K^ was shown to display a higher catalytic activity than Est1^A68V/T253P^, with a specific activity of 910 ± 42.0 U/mg, and the thermostability was lower than that of Est1^A68V/T253P^ over 50°C.

In a recent study, the crystal structure of Est119 complexed with ethyl lactate (EL) and lactic acid (LAC) was obtained (Figure 10(2)) (Kitadokoro et al., 2019). As expected, three Ca^2+^ ions were found in Est119; moreover, LAC and EL are located in the active site cleft with different positions. Based on the crystal structure, it was found that the substrate-binding cleft was a long narrow groove lined with F248, I217, H247, S169, W194, M170, Y99, T100, T102, S105, and N251. In the binding site, EL was stabilized by the H-bonds formed with H247 and water and the hydrophobic interaction with H168. In addition, LAC was regulated by the hydrophobic interaction with W194 and the H-bonds formed with water. The catalytic S169 is located at the bottom of the cleft.

Recently, a novel directional-path modification (DPM) strategy was developed to improve the performance of PET-degrading enzymes, which mainly contains the introduction of positively charged amino acids and binding groove remodeling (Chen et al., 2022). First, the PET binding path was identified. Then, to decrease steric hindrance in the binding site at the exit of the PET binding path, neutral short side-chain amino acids were introduced. At the same time, positively charged amino acids with shorter flexible side chains (e.g., Lys) were selected to optimize the surface positively charged area. These modifications led to the identification of 13 potential residue sites and construction of 23 target mutants, and the results showed that the four-point mutant (4Mz, H184S/Q92G/F209I/I213K) displayed the highest degradation rate of ~90%. The degradation efficiency was increased by approximately 30-fold compared with that of the wild type. Molecular simulations revealed that the PET binding modes of 4Mz were extended to the left side, which was estimated to be more conducive to improving PET degradation. This finding was also confirmed by positive vacuum electrostatics on the left-side subbinding site. When applied to LCC, Est119, and BhrPETase, the constructed mutants LCC^H218S/Y127G/F243I/S247K^, Est119^H248S/Q131G/F248I/I252K^ and BhrPETase^H184S/F93G/F209I/S213K^ all showed dramatically increased PET degradation efficiency, indicating the high efficiency and universality of the DPM strategy.

#### 2.2.6. Polyesterase from *Deinococcus maricopensis* (*Dm*PETase)

In a recent study, a novel thermophilic polyesterase from *Deinococcus maricopensis* (*Dm*PETase) was characterized (Makryniotis et al., 2023). *Dm*PETase, a phylogenetically distinct and thermophilic polyesterase, was found to display a similar overall structure with that of other bacterial cutinases, such as LCC^ICCG^ and *Is*PETase, and the catalytic triad is determined to be Ser185, His263 and Asp231. The optimal temperature for *Dm*PETase was 50°C lower than that of LCC^ICCG^, and display the highest catalytic efficiency toward *p*NPB. It is also capable of degrading various synthetic polymers, including PET, polyurethane, as well as four semi-crystalline aliphatic polyesters. On the whole, *Dm*PETase shows a much lower catalytic activities compared with those of LCC^ICCG^, for example against the PCL powder *Dm*PETase displayed a degrading approximately 70% of the tested materials compared with 98.7% obtained by LCC^ICCG^. However, at 50°C, it displays a comparable degrading activity compared with that of LCC^ICCG^ against the semi-crystalline sections of post-consumer PET bottles (crystallinity, 41%).

### 2.3. Cutinase from fungi

#### 2.3.1. Cutinase from *Humicola insolens* (HIC)

**Figure 11:**
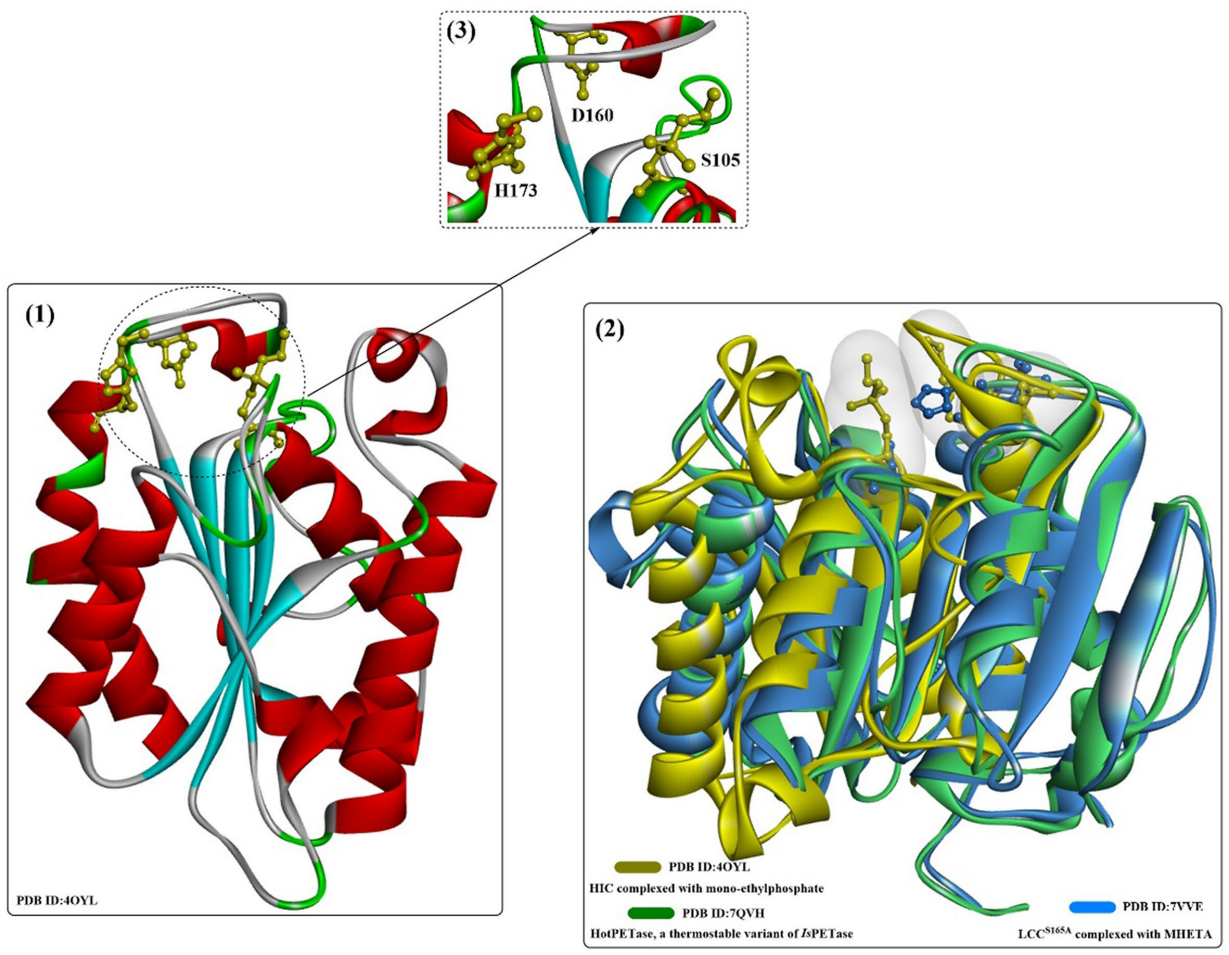
The structure and active site of HIC (1), the structure alignment of bacterial LCC (2, in blue) and *Is*PETase (2, in green), and the local detailed structure of the active site of HIC. [The hydrophobic regions of the substrate binding site are highlighted in white surface, and the catalytic triads are shown in ball-and-stick model in (3)].

The cutinase from the fungus *Humicola insolens* (HIC, Figure 11) is a single-domain protein that shows high hydrolytic activity and stability at 70°C (Carniel et al., 2021), and sequence analysis showed that HiC exhibits high sequence identities to FsC (56%) (Kold et al., 2014).

When used in moist-solid reaction mixtures instead of the typical dilute aqueous solutions or slurries, cutinase from *Humicola insolens* can directly depolymerize amorphous and crystalline regions of PET equally without any pretreatmentl a 13-fold higher space–time yield and a 15-fold higher enzyme efficiency were achieved than those reported in prior studies with high crystallinity material. Furthermore, this process shows 26-fold selectivity for terephthalic acid over other hydrolysis products (Kaabel et al., 2021).

In an earlier study, it was found that cutinase from *Humicola insolens* (HIC) could hydrolyse PET, especially the low-crystallinity (7%) film (~10-fold higher than biaxially oriented PET, 35% crystallinity) (Ronkvist et al., 2009). Moreover, HiC shows good thermostability and functions best from 70 to 80°C, and the HiC-catalyzed degradation of the *lc*PET film resulted in a 97 ± 3% weight loss only releasing the water-soluble TPA after 96 h at 70°C. Based on this finding, a novel synergistic chemoenzymatic hydrolysis of PET was developed to produce terephthalic acid (TA) from textile waste (Quartinello et al., 2017). First, conversion of PET into TA (purity, 85%) and small oligomers was achieved by chemical treatment (*T* = 250°C, *p* = 40 bar). Afterwards, the obtained oligomers were further hydrolysed by HiC, yielding ~6.5 mM TA after 6 h of incubation at 50°C (purity, 97%). This is a practical strategy for the efficient recycling of PET in an environmentally friendly way under neutral conditions, especially for promising catalysts displaying lower catalytic activities toward high-crystallinity PET.

Carniel et al. (2021) investigated the HiC-assisted hydrolysis of postconsumed PET (PC-PET) through controlling the pH and performing fractionation of enzyme feeding in stirred reactors. The results showed that pH variation plays a critical role in the HiC-catalyzed reaction, and a 0.86 pH variation (optimal pH 8.95 ± 0.20) finally led to a decrease of 32% in the total of final products. Compared with the tris-buffered reaction, the soluble hydrolysis products from the unbuffered HiC-catalyzed reaction with pH control were increased by 81% at 50°C (26.3 mM). Although enzyme feeding fractionation contributed little activity for the improved PET hydrolysis, an increase of 2.41-fold in the final degrading products released (97 mM) per protein amount was obtained when only half of the enzyme was loaded. These findings are important for the industrial PET biorecycling process.

Additionally, in a recent study, HiC-catalyzed PC-PET degradation was optimized (PET particle size, pH, ionic strength, and enzyme concentrations) for improved performance with experimental and mathematical modeling approaches (Eugenio et al., 2021). The results revealed that the PET particle size might play the most critical role in HIC-catalytic PET hydrolysis, and with a greater surface area (semicrystalline PC-PET), an 8.5-fold increased hydrolysing rate (1.7 mmol·L^−1^·h^−1^) was achieved. After 4 days at 70°C, 10.9 mmol·L^−1^ TPA was detected against the semicrystalline PET (0.075–0.25 mm). The thermostability of wild HiC is much higher than that of the other reported cutinases, the half-life of which was determined to be 110 h at 70°C and pH 7.0. These results indicated that HiC displays great potential as a promising biocatalyst for efficient PET biodepolymerization.

**Figure 12:**
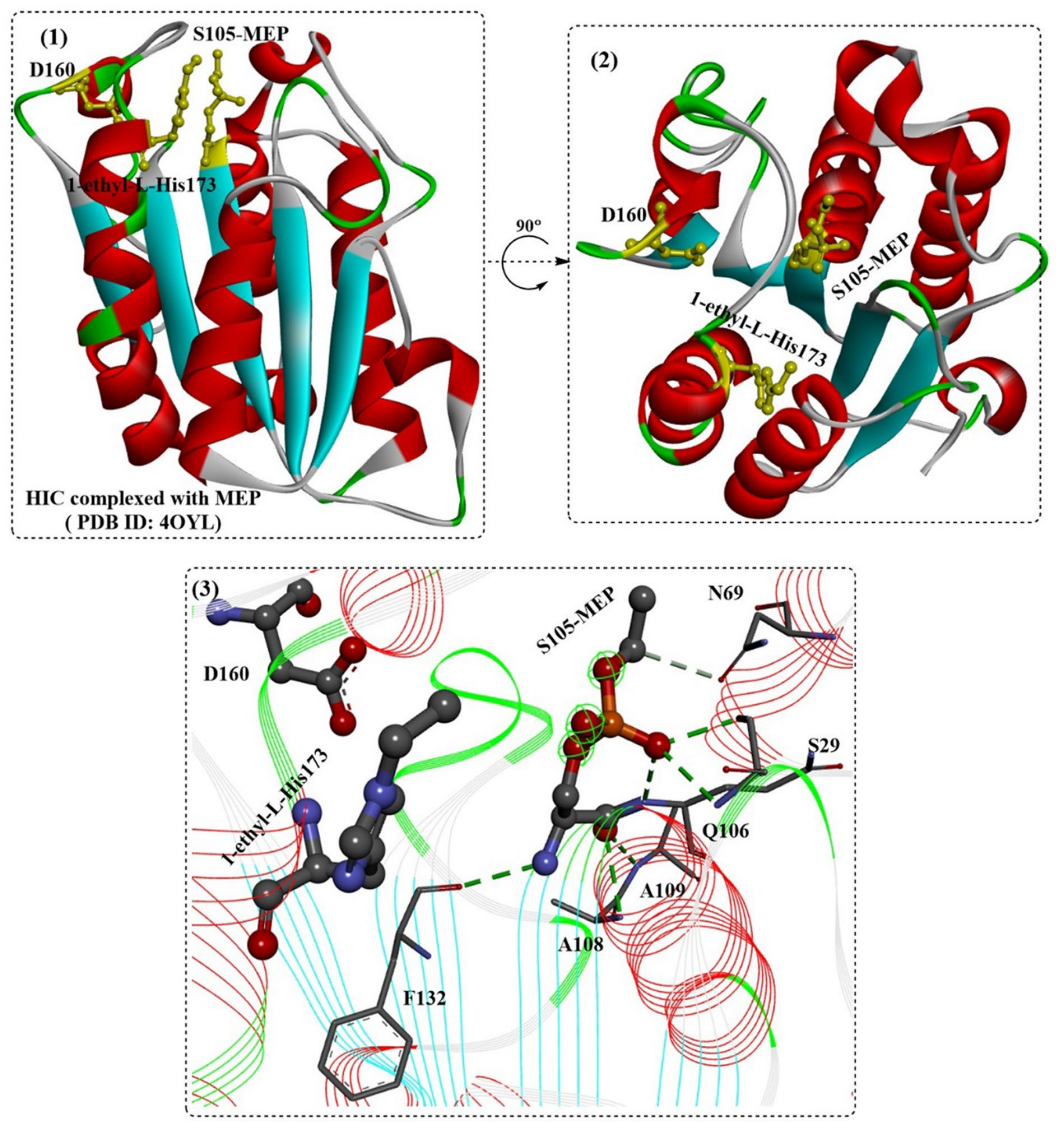
The structure of the binding mode of MEP in the active site of the HIC (1,2) and the local detailed interactions between MEP and HIC. [The catalytic triads are shown in ball-and-stick model, especially the ethylated H173, and the (or light) green dotted lines mean the formed H-bonds].

SDS might be the most widely applied anionic surfactant and can regulate the bioactivities of cutinases or cutinase-like PET-degrading enzymes. The interactions of cutinase from *Humicola insolens* (HIC) with anionic surfactants (SDS) were investigated in several studies (Nielsen et al., 2005; Kold et al., 2014). The protein could specifically bind SDS at a very low molar ratio of SDS/HiC, and amino acids 160–163 and 168–176 (near the active site) were determined to be able to directly interact with SDS by MD simulations. Kold et al. (2014) investigated the detailed molecular interactions between SDS and HiC based on thermodynamic and structural studies. Crystal structures showed that the binding mode of SDS is quite similar to the previously reported substrate analogs to cutinases (Figure 12(2)), and the binding affinity (*K*) was determined to be ~10^5^ M^−1^. In addition, mono-ethylphosphate (MEP) was found to covalently bind to S105 and could interact with F132, A108, A109, Q106, S28 and N69 by forming H-bonds (Figure 12(3)). Moreover, ethylated H173 was detected, probably resulting from the reaction with DNPP (diethyl p-nitrophenyl phosphate).

In a recent study, high-crystallinity PET in mixed PET/cotton textiles was reported to be directly and selectively depolymerized to TPA by using a commercial HIC (Novozym 51,032) and the Cellic CTec2 cellulases under moist-solid reaction conditions (Kaabel et al., 2023). Results revealed that TPA is the main degrading products with more than 40-fold selectivity over MHET after 7 days of static incubation at 55°C in the presence of HiC (0.65% w/w) and/or CTec2 (0.7% w/w), and 14.1% of TPA could be obtained when co-applied with CTec2. Interestingly, it was also found that the CTec2 enzymes were most efficient in the absence of HiC with up to 83.4% yield of glucose without any negative influence on the TPA yield.

#### 2.3.2. Cutinase from *Fusarium solani pisi* (FsC)

**Figure 13:**
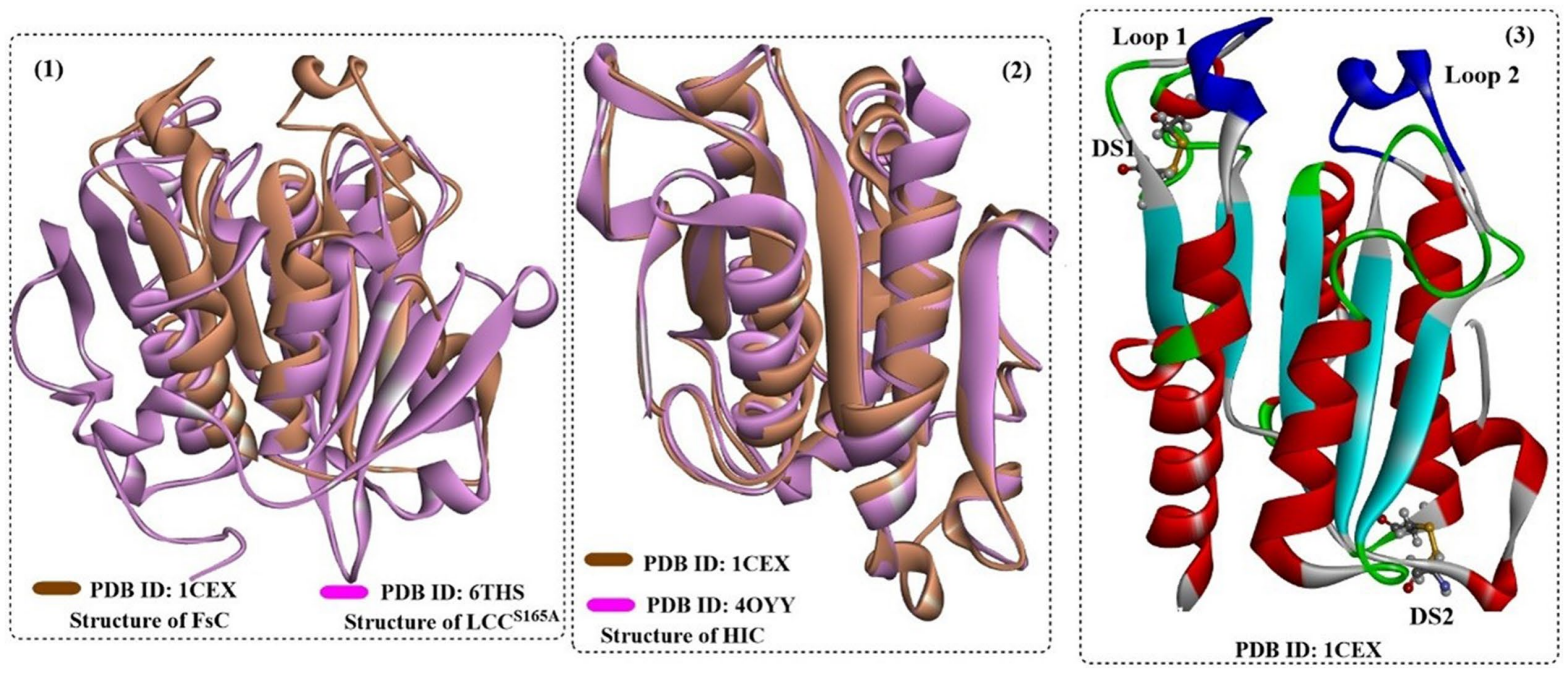
The structure alignment of FsC with LCC (1) and HIC (2) and the overall structure of FsC (3). [In (3), the formed two disulfide bonds are shown in ball-and-stick model and the typical loop1 and loop2 are highlighted in dark blue].

FsC has long been explored for the hydrolysis of several industrially important esters, insoluble PET polymer films and poly(ε-caprolactone) (PCL) (Ronkvist et al., 2009). FsC is also a single-domain protein consisting of 197 amino acids (~20.8 kDa). The overall structure of FsC is similar to that of Hic, which is different from the bacterial-derived cutinase as a single-domain protein (Figure 13(1,2)). FsC also contains 5 slightly twisted parallel-stranded β-sheets that are covered by 4 α-helices on each side (Figure 13(3)). Among them, 2 flexible loop domains (residues 80–90 and 182–189) and 2 disulfide bridges (DS1:Cys31 and Cys109, DS2:Cys171 and Cys178) are highly conserved and may be associated with substrate binding and the stabilization of the overall structures (especially DS1) (Matak and Moghaddam, 2009).

In a recent study, NMR spectroscopy was used to investigate FsC with PET-hydrolysing activity and the reaction, including the reaction conditions, interactions between FsC and BHET, and product release (Hellesnes et al., 2023). It was found that BHET binding is predominantly regulated by hydrophobic interactions (with adjacent polar residues and polar residues); in particular, a more extended binding cleft around G49 and G192 is needed. Given that FsC^L81A^ (improved by 4-fold) and FsC^L182A^ (improved by 5-fold) showed much higher hydrolytic activity on PET and polyamide 6,6 fibers, the enlarged binding site might more likely result in improved cutinase activity, as discovered by Araújo et al. (2007). The main product (80% of the products) from PET hydrolysis is MHET, and after ~400 min, the release of TPA was detected (Hellesnes et al., 2023). More importantly, it was found that the pH and pD conditions might regulate the relative amounts of degradation products, and the low degrading activity of FsC on PET is caused by poor substrate binding and the subsequent slow MHET hydrolysing activity. Previously, it was concluded that increasing accumulation of the final product EG around the active site reduced the overall flexibility of FsC (Groß et al., 2017), which certainly caused decreased thermostability and catalytic performance.

To further exploit the potential of FsC in the biodegradation of parabens, a novel biocatalyst (named SDFsC) was constructed by expressing FsC on the cell surface of *Sacchromycese cerevisiae* with Aga1p and Aga2p protein subunits and a glycosylphosphatidylinositol (GPI) attachment sequence (Zhu and Wei, 2019). Catalytic results indicated that SDFsC could be used for the efficient removal of parabens with *K*_m_ values of 3.1 ± 0.5 mM (for *p*NPA) and 0.67 ± 0.15 mM (*p*NPB); thus, the catalytic performance of SDFsC was slightly affected. In addition, the side chain structures of parabens play a critical role in the degradation rates, and parabens with relatively long alkyl or aromatic side chains (propylparaben, butylparaben and benzylparaben) were more efficiently degraded, with efficiencies of 89, 97 and 93%, respectively, after 24 h at 30°C. Moreover, SDFsC showed excellent reusability with 93% residual enzyme activity after being used for paraben degradation 6 times. Altogether, it is believed that biocatalysis based on the enzyme cell surface display would be a green and efficient alternative for efficient PET biodegradation.

#### 2.3.3. Cutinase from *Alternaria brassicicola* (AbC)

In 1997, two distinct serine esterases with cutinolytic activity (52 kDa and 26 kDa) were discovered (Trail and Köller, 1993), and a cutinase-encoding gene, *CUTAB1*, was then identified from an *A. brassicicola* cDNA library (Yao and Kller, 1994).

The cutinase CUTAB1 from *Alternaria brassicicola* (corresponding to the cutinase from the *A. brassicicola* culture) was successfully heterologously expressed in *Pichia pastoris* (Koschorreck et al., 2010). It displayed the highest enzymatic activity at 40°C and pH 7–9, and similarly, the chain lengths of different substrates showed significant effects on the hydrolysing activity; the highest catalytic activity was toward tributyrin and the lowest activity was toward *p*-nitrophenyl palmitate (*p*-NPP). In contrast to the findings for FsC (Hellesnes et al., 2023), the mutants AbC^L80A^, AbC^L181A^ and AbC^I183A^ showed a significant decrease in specific activity toward *p*-NPB and *p*-NPP. In contrast, the specific activity of AbC^A84F^ against *p*-NPP was significantly improved by ~10-fold. This strange phenomenon was thought to be associated with increased interactions with longer chain and hydrophobic substrates caused by the bulkier and more hydrophobic phenylalanine.

#### 2.3.4. Cutinase from *Aspergillus fumigatus* (AfC)

Two cutinase genes from *Fusarium solani* (FsC) and *Aspergillus fumigatus* (AfC) were also successfully expressed in *Pichia pastoris* X33 (Ping et al., 2017). The results showed that FsC and AfC display significantly different optimal catalytic temperatures (40°C and 60°C, respectively) but similar pH values (7.5 and 8.0, respectively). This illustrated the superior thermostability of AfC. Both FsC and AfC can hydrolyse *p*-nitrophenyl substrates with different carbon chain lengths, and *p*NPD and *p*NPH were the preferred substrates for FsC and AfC, respectively. Moreover, AfC could completely hydrolyse the PCL film.

#### 2.3.5. Cutinase from *Aspergillus oryzae* (AoC)

*Aspergillus oryzae* is a well-known filamentous fungus that has long been used in traditional microbial fermentation and the food industry. Recently, the fungus has attracted much attention for the biodegradation of poly(butylene succinate) (PBS) and poly(butylenes succinate-co-adipate) (PBSA) with the successful identification of a PBS-degrading enzyme (AoC) (Maeda et al., 2005). AoC (~21.6 kDa) displayed the highest catalytic activity at 55°C and could remain stable under a wide pH range (pH 6.0–11.0).

**Figure 14:**
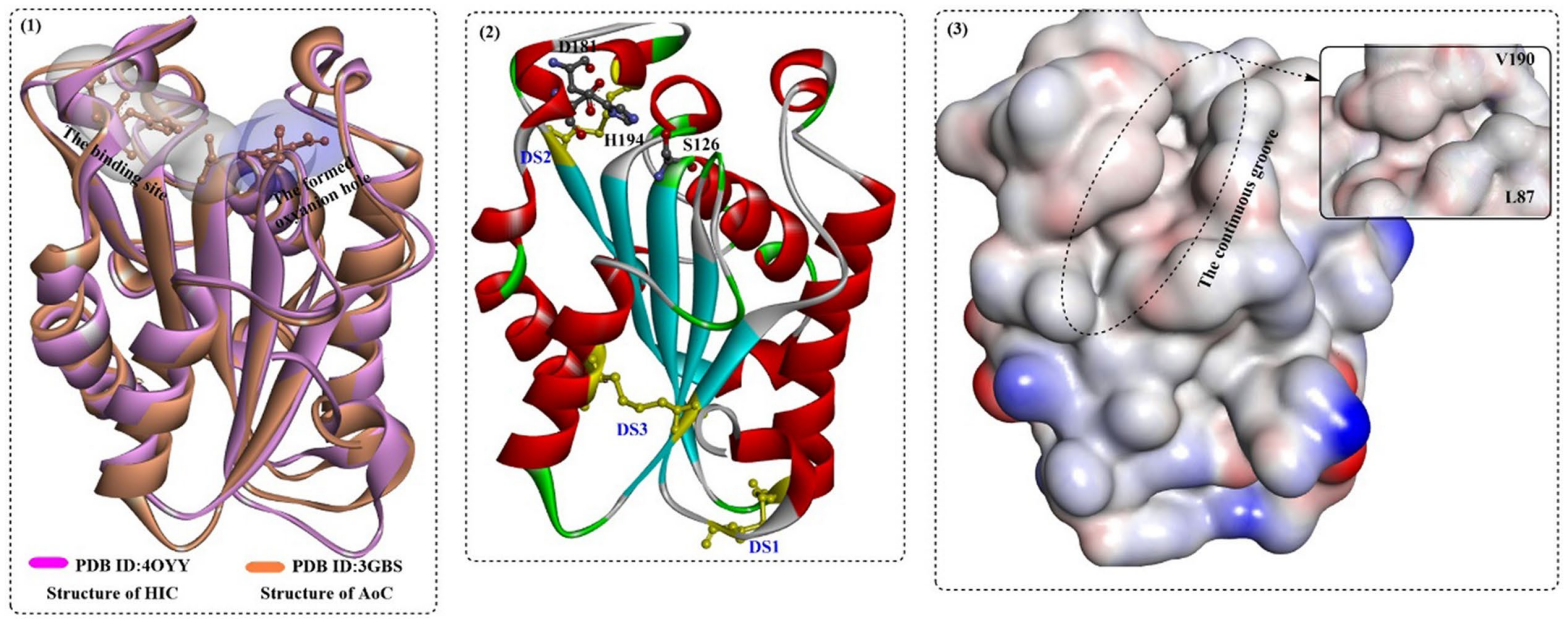
The structure alignment of AoC with HIC (1) and the special features of AoC (2,3). [In (1), the substrate binding site and the formed oxyanion hole are show in white and light blue surface; in (2), the formed three disulfide bonds and the catalytic triads are shown in in ball-and-stick model, and in (3) the hydrophobic surface of the HIC protein is displayed especially the continuous groove for substrate binding].

In a recent study, the biological properties of AoC were investigated, and the structure–activity relationship was analyzed based on the crystal structure (Liu et al., 2009). AoC showed better thermostability with a *T*_m_ value of 59°C. AoC showed a preference for *p*NPV with a *K*_m_ value of 0.04 ± 0.01 μM and displayed the highest catalytic efficiency toward *p*NPB and *p*NPV with *k*_cat_/*K*_m_ values of 3.49 ± 0.51 μM^−1^ min^−1^ and 3.32 ± 0.74 μM^−1^ min^−1^, respectively. At 40°C and pH 8.0, poly ε-(caprolactone) (PCL) was almost completely degraded (87% PCL) within 6 h; however, only 30% degradation was achieved catalyzed by FsC. The structure (Figure 14(1)), which is similar to that of FsC and HiC, contains a typical α/β fold characterized by a central β-sheet of 5 parallel strands; this sheet is surrounded by 10 α-helices with a highly conserved catalytic triad S126, D181, and H194. In addition, 3 disulfide bonds were identified, including DS1 between Cys37 and Cys115, DS2 between Cys177-Cys184, and DS3 between Cys63-Cys76. Among them, DS3 is unique to the cutinases from the *Aspergillus* family connecting helix 2 to strand 2 of the central β-sheet, which might lead to improved thermoactivity of AoC. Different from FsC and HiC, an extended groove was discovered around the substrate binding site that regulates the orientation of polymeric chains (Figure 14(3)), which resulted in a much higher hydrolysing activity of AoC toward the longer chain substrates. Furthermore, residues L87 and V190, similar to the “gate keepers,” may be associated with substrate recognition, especially the different alkyl chains.

To further improve thermostability, Shirke et al. investigated the thermal inactivation mechanisms of AoC (Shirke et al., 2017). First, it was revealed that glycosylation plays a critical role in the improved thermostability of *Thiellavia terrestris* cutinase (TtC) expressed in *Pichia pastoris* by inhibiting its thermal aggregation. In view of this finding, kinetic models of cutinase thermal inactivation were created to account for thermal aggregation. However, the models also indicated that the glycosylation of TtC is helpful for the improved kinetic stability (5-15-fold) by inhibiting thermal aggregation at 61°C. Then, one glycosylation site (L185N) was designed and incorporated into AoC, which completely inhibited the thermal aggregation of AoC with a 4-fold improvement in thermostability. However, it should be noted that the undesirable catalytic efficiency of AoC^L185N^ was lower (~70%) than that of wild-type AoC, and this may be avoided by rationally designed glycosylation sites.

Recently, Lewatit VP OC 1600 was used as the macroporous support for the immobilization of AoC, HIC and TfC for industrial applications of cutinases (Su et al., 2018). The results demonstrated that HiC displays the highest solvent tolerance with increased polarity, and TtC shows the best thermostability at 80°C. Furthermore, in nonane, the three obtained catalysts could maintain ~64% residual enzyme activity even at 90°C, which reveals that this strategy is efficient in improving the organic solvent tolerance.

In a study, the abovementioned cutinases were investigated in detail (Baker et al., 2012). HiC was shown to exhibit the highest stability with significantly improved poly(ε-caprolactone) hydrolysing activities at high temperatures under the tested conditions. Additionally, at low pH conditions, HiC could maintain the overall structure with little affected activity, but AbC exhibited the largest loss in structure. Similarly, FsC and AbC were found to be the least stable at high temperatures.

#### 2.3.6. Metagenome- and proteomics-derived PET hydrolase

**Figure 15:**
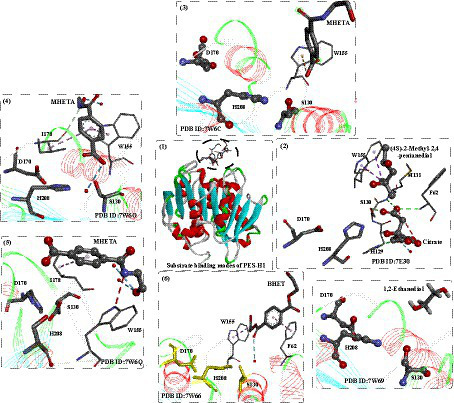
The binding modes of substrate analogs and degradation intermediates in the active site of PES-H1 (1), and the local detailed interactions modes of different substrates in the binding site (2–7). (2: detailed interactions of (4S)-2-Methyl-2,4-pentanediol in the active sits; 3: detailed interactions of MHETA in the active sits; 4: detailed interactions of MHETA in the active sits; 5: detailed interactions of BHET in the active sits; 6: detailed interactions of MHETA in the active sits; 7: detailed interactions of 1,2-Ethanediol in the active sits).

Recently, two highly similar thermophilic PET hydrolases (PES-H1 and PES-H2) were identified from a compost metagenome library, and PES-H1 and PES-H2 differ in only four residues (A/E1, L/F209, D/N232, and S/A254) (Sonnendecker et al., 2022). Although they contain quite similar amino acid sequences, PES-H1 displayed a much higher hydrolytic activity on amorphous PET films, even higher than that of the wild-type LCC under identical conditions. To investigate the catalytic mechanism, high-resolution crystal structures of PES-H1 complexed with various PET substrates were obtained, including citrate [PDB ID: 7E30, with 4-(2-hydroxyethylcarbamoyl) benzoic acid (MHETA), PDB ID:7W6C, 7W6O, and 7W6Q] and with bis(2-hydroxyethyl) terephthalate (BHET, PDB ID: 7W66) (Pfaff et al., 2022). Regarding the structures, PES-H1 adopts the canonical α/β-hydrolase fold consisting of 9 β-sheets and 10 α-helices and exhibits highly conserved features of the α/β-hydrolase superfamily similar to LCC. Overall, the binding modes of the different ligands in the active sites are quite different but similar to those observed for HEMT bound to *Is*PETase ^R103G/S131A28^ and MHET bound to the LCC ^ICCG^. Given that the multiple noncatalytic intermediate binding modes (e.g., MHETA) far from S130 are less likely to undergo nucleophilic attack, enzyme-mediated PET hydrolysis involves the dynamic reorientation of polymer chains in the substrate-binding site. The effect of W155 was also identified (Figure 15(2–7)), which is critical for PET binding and stabilization. Especially, from Figure 15(2–4), the different conformations of MHETA were obtained, which clearly revel the MHET degrading process. MHETA located away from the catalytic triad is stabilized by hydrophobic residues, including F62, I178, L209, M131 and F62. In addition, the TPA moiety is in the vicinity of F62 rather than close to the catalytic triads. Moreover, the special binding mode of MHETA located at the surface of PES-H1 (Figure 15(4)) indicates that for PET hydrolysis, it might first bind at the surface and be transferred into the deeper active site cavity.

Given the crystal structures and the MD simulations, mutational hotspots were identified for rational engineering of PES-H1, such as the R204C/S250C variant, R6C/S49C variant, L92F/Q94Y, and L92F/Q94Y/R204C/S250C variant. The results showed that mutant PES-H1^L92F/Q94Y^ displayed a 1.8°C higher *T*_m_ and could almost completely degrade the Gf-PET film after 24 h at 72°C, with the highest weight loss of 37.1 mg (62.4%). It outperforms the promising LCC ^ICCG^ mutant in the biohydrolysis of low-crystallinity (13%) PET powder with 2.2-fold more degradation products. It was believed that the enhanced activity was caused by the “aromatic tunnel” (substitutions of L90F) effect streaming the efficient binding of PET chains to the active site (Cui et al., 2021).

## 3. Enhancing PET hydrolytic activity by constructing fusion proteins

As a surface erosion process, low binding affinity with PET, as a critical factor, has been found to significantly restrict the further industrial application of various PET hydrolases. In addition to modulating protein evolution, the strategy involving constructing fusion proteins has also been proven to be effective in enhancing the binding affinity of enzymes and substrates with hydrophobic surface binding modules (Ribitsch et al., 2013; Várnai et al., 2014; Ribitsch et al., 2015).

Carbohydrate-binding modules (CBMs) are small noncatalytic protein domains that usually function as a part of modular enzymes (e.g., carbohydrate-active enzymes, CAZymes), which can enhance the binding affinity between enzymes and substrates (Armenta et al., 2017; Weber et al., 2019). Consequently, CBMs have been used as efficient affinity tags for protein immobilization owing to their high adsorption capacity for solid substrates (Oliveira et al., 2015; Zhou et al., 2020). Based on the topology of the ligand-binding site, CBMs can be classified into types A, B (*endo*-type), or C (*exo*-type) (Gilbert et al., 2013). Type A CBMs display a high ability to bind crystalline polysaccharides (e.g., cellulose and chitin) through hydrophobic interactions regulated by conserved aromatic triplets and are therefore potential candidates for PET-binding peptides (Zhang et al., 2013; Liu et al., 2022).

Weber et al. (2019) identified a novel PET-binding CBM and explored the CBM-PET interactions. First, a semiquantitative PET surface affinity assay was developed to detect CBMs bound to PET films. Then, eight CBMs from the carbohydrate active enzymes database^4^ (Drula et al., 2022) were screened for PET binding, and the results showed that *Ba*CBM2 (GenBank accession numbers: ACQ50287/MK349005) possessed the strongest affinity toward PET. To investigate the CBM-PET interactions, molecular dynamics (MD) simulations identified an aromatic triad (Trp9/Trp44/Trp63) on the peptide surface that was stabilized by π-stacking interactions and hydrogen bonds. This was further verified by tryptophan quenching experiments and alanine point mutations; moreover, the strength of PET binding of CBMs was largely determined by the ratio of hydrophobic to polar contacts at the interface.

In a recent study, fusion of different CBMs to the C-terminus of LCC^ICCG^ was reported to improve the PET hydrolysing activities, including a ChBD from *Chitinolyticbacter meiyuanensis* SYBC-H1, the CBM from *Hypocrea jecorina*, the polyhydroxyalkanoate binding modules (PBM) from *Alcaligenes faecalis* and *Trichoderma hydrophobins* HFB4 (Xue et al., 2021). The results showed that LCC^ICCG^-ChBD displayed a low *K*_m_ value (131.9 μM) but a much higher *k*cat/*K*_m_ (0.343 s^−1^/μM) compared to LCC^ICCG^ (171.8 μM, 0.178 s^−1^/μM), indicating better substrate affinity to amorphous PET film (GF-PET, crystalline 6.7%). The adsorption of LCC^ICCG^-ChBD to GF-PET films revealed that a maximum adsorption efficiency of 30.2% was achieved after 70 min of incubation at 40°C. For *p*NPB hydrolysis, LCC^ICCG^-ChBD showed a lower *K*_m_ and slightly lower catalytic efficiency (*k*_cat_/*K*_m_, 0.404 s^−1^/μM) than LCC^ICCG^ (0.534 s^−1^/μM). However, for the ground GF-PET powder, depolymerization degrees of 87.5 and 98.5% were achieved with LCC^ICCG^-ChBD and LCC^ICCG^-CBM at 65°C, respectively, which are 19.6 and 30.6% higher than those obtained with LCC^ICCG^.

In a similar study, to enhance the binding affinity of LCC^ICCG^ with PET, *Tr*CBM and *Cf*CBM were rationally selected from the CAZy database to construct fusion proteins with LCC^ICCG^ (Chen et al., 2023). Additional auxiliary domains *Tr*CBM (PDB ID: 1AZ6) from *Trichoderma reesei* and *Cf*CBM from *Cellulomonas fimi* were obtained from the CAZy database^5^ (Drula et al., 2022). *Cf*CBM and *Tr*CBM belong to the type A carbohydrate-binding modules (CBMs), which can bind hydrophobic substrates (e.g., chitin) through hydrophobic interactions. Then, blind molecular docking and molecular dynamics were applied to identify potential binding positions of 4-PET, and the results showed that five positions (2 for *Tr*CBM and 3 for *Cf*CBM) were favorable for the binding of PET-4 to CBMs, which occurred mainly through van der Waals interactions, conventional hydrogen bonding and pi-pi interactions. Then, the fusion proteins LCC^ICCG^-*Tr*CBM and *Cf*CBM-LCC^ICCG^ were generated and characterized. The results showed that the optimal temperatures of LCC^ICCG^-*Tr*CBM and *Cf*CBM-LCC^ICCG^ were further increased to 70 and 80°C, respectively. Compared with LCC^ICCG^ and *Cf*CBM-LCC^ICCG^, LCC^ICCG^-*Tr*CBM displayed the strongest thermal stability with 70.1% residual activity at 50°C for 24 h. *Cf*CBM-LCC^ICCG^ and LCC^ICCG^-*Tr*CBM were found to more favorably bind to PET films, increasing by 1.4- and 1.3-fold in comparison to LCC^ICCG^. The total products released from *Cf*CBM-LCC^ICCG^ were shown to be 24.2% higher than those from LCC^ICCG^. This study clearly describes the significant contributions achieved that enhance the binding ability of LCC^ICCG^ to PET films and significantly improve the degradation ability of the crystalline region of PET materials by constructing fusion proteins.

Rennison et al. (2023) investigated the binding of *Ba*CBM2 for PET in detail and showed that the binding affinity is highly associated with temperature and crystallinity. With the increase in tested temperatures, the general trend of *K*_d_ increased first after declining. At ~40°C, a maximum *K*_d_ of 408 nM for *Ba*CBM2 was detected, showing an obviously lower affinity for this PET substrate. At low temperatures, the increased binding affinity might be caused by a gain in entropy caused by dehydration of the binding surface; nevertheless, at higher temperatures, it might result from an increase in crystalline regions on the PET surface. At 20°C, a clear trend is observed in which the affinity increases at higher crystallinity, and a significant increase in affinity was seen as ~20% bulk crystallinity. This indicated that *Ba*CBM2 might bind preferentially to crystalline areas on the PET surface. MD studies indicated that the binding is mainly driven by the functions of critical residues W9, W44 and W63 through a gain in entropy, which is caused by the dehydration of the binding surface. We believe that fusing a polymer-binding module to the PET hydrolase is a promising strategy to streamline the enzymatic hydrolysis of PET, however the binding capacity, affinity, and thermostability should be improved to satisfy the needs of industrial operation.

## 4. Conclusion

It has absolutely been accepted that biodegradation may create new opportunities for recycling PET materials. Research on PET biodegradation is still ongoing, and while progress has been achieved, the following challenges remain in developing efficient and scalable methods for biodegrading PET. (1) The relatively slow degradation rates: PET is a highly stable and resistant polymer, and it requires a long time for natural biodegradation processes to break it down. Enzymatic and microbial methods are also slow, and full biodegradation of PET can last weeks or even months. (2) Complexity of PET materials: PET is often used in complex products, such as multilayer packaging or textiles, which can make it more difficult to biodegrade. In some cases, only certain layers or components of the product may be biodegradable, while others are not. (3) Limited understanding of biodegradation mechanisms: While there has been significant progress in determining the mechanisms of PET biodegradation, much remains unknown. (4) Compatibility with recycling: Biodegradation of PET may pose challenges with traditional recycling methods, which often require separation of different types of plastics. (5) Cost: Developing and implementing effective and scalable methods for PET biodegradation can be expensive, which may limit their adoption by industry and governments. Overall, while challenges remain in developing effective and scalable methods for PET biodegradation, the potential benefits are significant and would certainly create new opportunities for sustainable materials management.

## Author contributions

BS: Writing – original draft, Writing – review & editing. TW: Conceptualization, Supervision, Validation, Writing – original draft, Writing – review & editing. JF: Data curation, Writing – original draft. ZH: Data curation, Writing – original draft. TS: Data curation, Writing – original draft. ZL: Resources, Software, Writing – original draft. FL: Conceptualization, Funding acquisition, Writing – original draft. YZ: Supervision, Validation, Writing – original draft.

## Funding

The author(s) declare financial support was received for the research, authorship, and/or publication of this article. The authors express their gratitude to the Research Fund for Academician Lin He New Medicine (No. JYHL2019MS12), the Supporting Fund for Teachers’ research of Jining Medical University (No. JYFC2019KJ037), the Shandong Provincial University Youth Innovation Team, China (No. 2022KJ102), and the National Natural Science Foundation of China (No. 32000194).

## Conflict of interest

The authors declare that the research was conducted in the absence of any commercial or financial relationships that could be construed as a potential conflict of interest.

## Publisher’s note

All claims expressed in this article are solely those of the authors and do not necessarily represent those of their affiliated organizations, or those of the publisher, the editors and the reviewers. Any product that may be evaluated in this article, or claim that may be made by its manufacturer, is not guaranteed or endorsed by the publisher.

## Footnotes

^1^ https://pazy.eu/doku.php

^2^ https://www.carbios.com/en/

^3^ https://doi.org/10.21203/rs.3.rs-2465520/v1

^4^http://www.cazy.org/, CAZy database.

^5^ http://www.cazy.org/
